# Spermine increases acetylation of tubulins and facilitates autophagic degradation of prion aggregates

**DOI:** 10.1038/s41598-018-28296-y

**Published:** 2018-07-03

**Authors:** Kanchan Phadwal, Dominic Kurian, Muhammad Khalid F. Salamat, Vicky E. MacRae, Abigail B. Diack, Jean C. Manson

**Affiliations:** 1 The Roslin Institute & R(D)SVS, University of Edinburgh, Easter Bush, Midlothian, EH25 9RG UK; 2 Centre for Dementia Prevention, University of Edinburgh, Edinburgh, UK; 3 Edinburgh Neuroscience, University of Edinburgh, Edinburgh, UK

## Abstract

Autolysosomal dysfunction and unstable microtubules are hallmarks of chronic neurodegenerative diseases associated with misfolded proteins. Investigation of impaired protein quality control and clearance systems could therefore provide an important avenue for intervention. To investigate this we have used a highly controlled model for protein aggregation, an *in vitro* prion system. Here we report that prion aggregates traffic via autolysosomes in the cytoplasm. Treatment with the natural polyamine spermine clears aggregates by enhancing autolysosomal flux. We demonstrated this by blocking the formation of mature autophagosomes resulting in accumulation of prion aggregates in the cytoplasm. Further we investigated the mechanism of spermine’s mode of action and we demonstrate that spermine increases the acetylation of microtubules, which is known to facilitate retrograde transport of autophagosomes from the cellular periphery to lysosomes located near the nucleus. We further report that spermine facilitates selective autophagic degradation of prion aggregates by binding to microtubule protein Tubb6. This is the first report in which spermine and the pathways regulated by it are applied as a novel approach towards clearance of misfolded prion protein and we suggest that this may have important implication for the broader family of protein misfolding diseases.

## Introduction

Accumulation and aggregation of misfolded proteins are hallmarks of neurodegenerative disorders, such as Alzheimer’s (AD), Parkinson’s, Huntington’s and prion diseases. These diseases many of which are strongly linked with age are debilitating and largely untreatable conditions. Considerable research has been directed towards understanding common pathological mechanisms that might be amenable to therapeutic intervention. Enhancing the pathways responsible for aggregate clearance such as autophagy is emerging as an important therapeutic approach in these disorders.

Autophagy is a highly conserved cellular degradation mechanism where the double membrane autophagosomes engulf the unwanted organelles, aggregates or cytoplasmic contents and pass them over to single membrane lysosomes for degradation. Fusion of autophagosomes with lysosomes forms a hybrid vesicle compartment called an autolysosome, these autolysosomes can be visualised by blocking the degradation via lysosomes. For healthy functional autophagic machinery all the elements of the pathway play a crucial role i.e. autophagosome formation, maturation of autophagosomes, engulfment of the cargo, trafficking of the autophagosomes, fusion of autophagosomes with lysosomes, lysosome numbers and rate of fusion of these vesicles. Compromise in any one on its own or together with other elements of the pathway leads to the failure of the autophagic machinery which can lead to plethora of pathophysiological defects^1^. It has been demonstrated that suppression of autophagy in neural cells leads to accumulation of ubiquitinated protein aggregates and subsequent neurodegeneration in mice^2,3^. Additionally upregulation of the bulk protein degradation process of autophagy has been shown to clear disease-associated proteins, such as mutant alpha-synuclein, ataxin-3, tau, huntingtin, TDP-43 and disease associated prion protein (PrP^Sc^) in various *in vivo* and *in vitro* models^4–9^.

Prion diseases provide a highly controlled and well-defined model system to study the course of the neurodegenerative disease process both *in vivo* and *in vitro*. Prion diseases are caused by the conversion of a normal cell-surface glycoprotein PrP^C^ to a misfolded protease resistant form, PrP^Sc^, which accumulates in the CNS leading to neurotoxicity and clinical disease. Disease associated prion protein can inhibit the 26S proteasome leading to impairment of the ubiquitin-proteasome system (UPS)^10^. In the absence of a functional UPS, the autophagosomal-lysosomal pathway is known to target insoluble protein aggregates for degradation^11^. Inducing autophagy using trehalose^12^, lithium^13^, rapamycin^8^ resveratrol^14^ and celecoxib derivatives AR-12^15^ has shown protection against prion infectivity in *in vitro* model systems. Furthermore, treatment with rapamycin and resveratrol has shown delayed disease onset in *in vivo* prion models^8,14^, although the precise mechanisms remain to be determined.

Likewise, polyamines spermine and spermidine are known inducers of autophagy and their synthesis and metabolism is inherent to the eukaryotic system. In the polyamine biosynthetic pathway, spermidine is a precursor of spermine and is a known promoter of longevity^16,17^. Spermidine has also been shown to prevent sporadic prion formation in yeast prion models^18^. Both enhancing longevity and protection against de novo formation of yeast prions by spermidine are shown to be autophagy dependent. Out of the several polycation tested for effectively eliminating PrP^Sc^, dextran-spermine a cationic polysaccharide version of spermine was determined to be most potent antiprion agent^19^. Spermine has also been shown to be the most effective inhibitor of nucleic acid induced polymerisation of human recombinant prion protein^20^ and is a known inducer of autophagy^21^. Given the enhanced efficacy of spermine over spermidine we investigated if and how spermine can clear prion aggregates.

We used prion infected cell cultures as a highly controlled model of chronic neurodegeneration to show that spermine at a physiological dosage^22^ can not only clear the prion aggregates but can also reduce cellular neurotoxicity by reducing the levels of cytosolic reactive oxygen species, ROS. This clearance of prion aggregates is autophagy dependent. Furthermore, treatment with spermine enhances the expression of microtubule proteins required for efficient retrograde transport of autophagic vesicles from the cellular periphery to the lysosomes in the cytoplasm. Finally, we report how spermine treatment targets prion aggregates to autophagosomes by binding with a microtubule protein Tubb6. Our results suggest multiple effects of spermine in the prion protein aggregation model. Its effect on enhancing autophagy, aggresome formation and acetylation of microtubules make it a potential therapeutic target for a broad family of protein misfolding diseases.

## Results

### Prion aggregates traffic via autolysosomes in SMB.s15 cells

**Figure 1:**
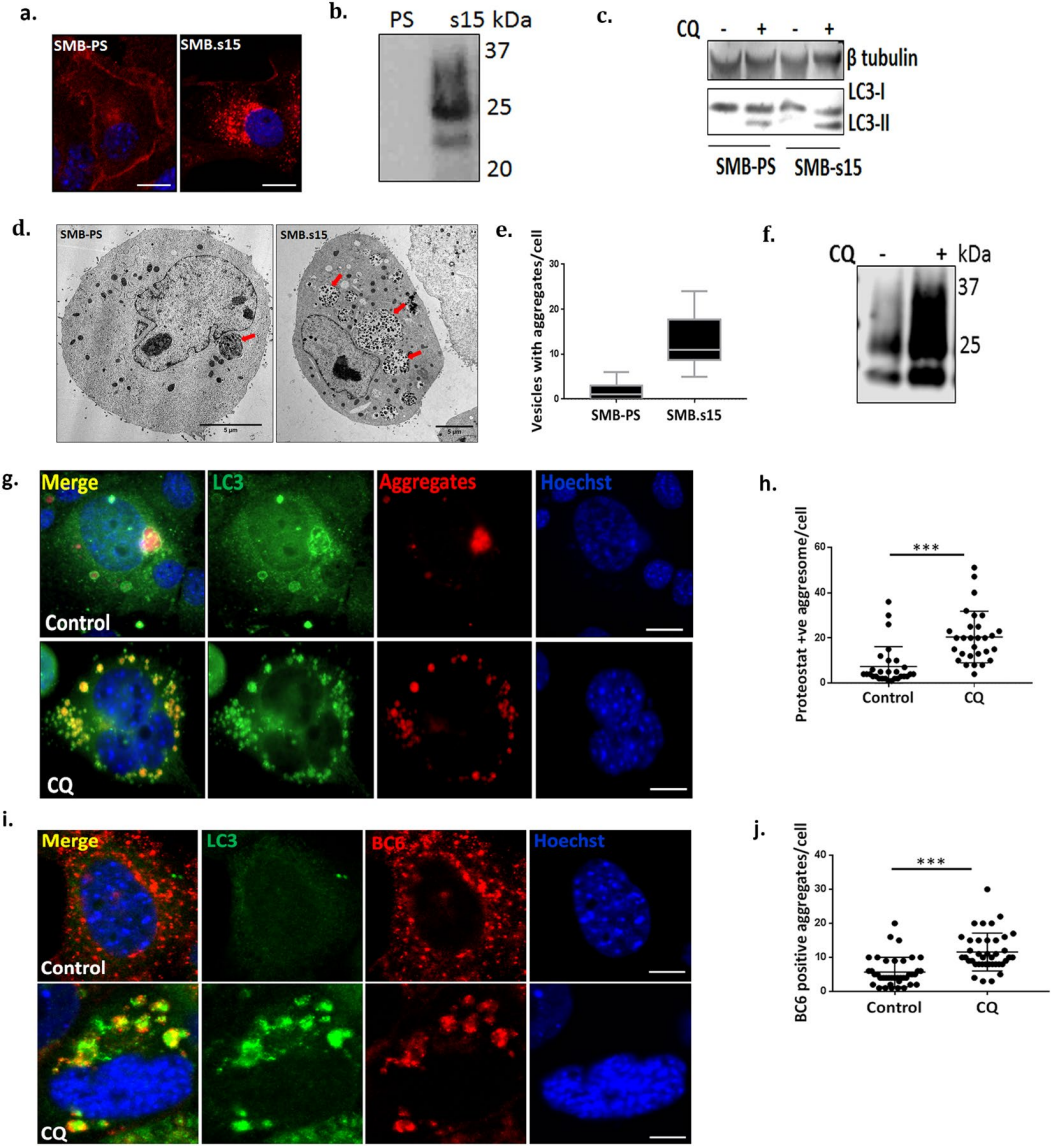
Prion aggregates traffic via autolysosomes in SMB.s15 cells. All the experiments were performed either with or without 10 μM CQ. (**a**) Immunofluorescence staining using anti PrP antibody BC6 in SMB-PS and SMB.s15 cells (N = 3). (**b**) Representative immunoblot shows PK resistant PrP^Sc^ in SMB.s15 cells (N = 2). (**c**) Representative immunoblot for LC3 and β-tubulin in SMB-PS and SMB.s15 cells, lane 2 & 4 were treated with CQ (N = 3). (**d**) Representative TEM images of SMB-PS and SMB.s15 after CQ treatment, red arrows indicate vesicles with aggregates (N = 2). (**e**) Increased number of vesicles with aggregates in SMB.s15 cells. All SMB.PS cells imaged with TEM showed minimal aggregates (0–5/cell) whereas all SMB.s15 cells images showed increased number of aggregates (5–24/cell). (**f**) WB for PK resistant PrP^Sc^ after CQ treatment in SMB.s15 cells (N = 3). (**g**) Representative image of proteostat dye (aggregates) and LC3 immunofluorescence staining of SMB.s15 cells after CQ treatment compared to untreated control cells (N = 3). (**h**) Significantly increased LC3 stained proteostat positive aggregates in SMB.s15 cells on CQ treatment. (**i**) Representative image of PrP^Sc^ and LC3 immunofluorescence staining of SMB.s15 cells after CQ treatment compared to untreated control cells (N = 5). (**j**) Significantly increased LC3 stained PrP^Sc^ aggregates in SMB.s15 cells on CQ treatment. Scale bar = 10 μm. Respective full length blots are shown in Supplementary Fig. 3. Abbreviations used Chloroquine (CQ), Transmission Electron Microscopy (TEM), proteinase K (PK).

We used the scrapie mouse brain (SMB) cell line infected with prion aggregates PrP^Sc^ (SMB.s15) and without prion aggregates (SMB-PS)^23^ for our study. We confirmed the presence of proteinase K (PK) resistant disease associated PrP^Sc^ both by immunostaining and immunoblotting with anti-prion antibody BC6. PrP^Sc^ bands were observed only in SMB.s15 cells but not in SMB-PS cells (Fig. 1a,b). We assessed both cell lines for basal levels of autophagy by measuring the conversion of autophagosome marker LC3-I to LC3-II by western blot (WB) after treatment with and without 10 μM of chloroquine (CQ) for 16 hrs. Increased conversion of LC3-I to LC3-II by blocking the autophagic flux with CQ is an indicator of enhanced autophagosome formation. Furthermore, incubating the cells with 10 μM CQ for 16 hrs results in the build-up of acidic vesicles such as lysosomes, autophagosomes and autolysosomes^24^. We observed an increased expression of LC3-II in SMB.s15 cells when compared to SMB-PS by WB. However, a complete conversion of LC3-I to LC3-II was not observed after CQ treatment (Fig. 1c), possibly indicating insufficient mature autophagosomes. Further analysis of the SMB cell lines by transmission electron microscopy (TEM) showed a clear accumulation of aggregate like bodies inside vesicles in the SMB.s15 cells but not in the SMB-PS after CQ treatment (Fig. 1d,e). These vesicles were observed with single membranes indicating that they may be autolysosomes. SMB.s15 cells also showed the presence of increased PrP^Sc^ on CQ treatment on WB (Fig. 1f). All these experiments established SMB.s15 as a convincing model of protein aggregation enabling us to investigate the role of autophagy in clearance of these aggregates.

We further confirmed whether the vesicles observed under TEM are autolysosomes and whether the aggregates inside these vesicles are PrP^Sc^ aggregates. Cells were treated with CQ to block the degradation via lysosomes. 5 M guanidine isothiocyanate treatment after fixation was used to expose the PrP^Sc^ epitopes^25^. Cells were stained with either proteostat dye (PS), which binds to protein aggregates, and the autophagy marker LC3 (Fig. 1g,h) or anti-prion antibody BC6 and LC3 (Fig. 1i,j). Staining of aggregates both with the PS dye and BC6 antibody showed the presence of punctate LC3 staining surrounding aggregates on CQ treatment, thus suggesting the presence of mature autophagosomes or autolysosomes around PrP^Sc^ aggregates. Together these results clearly indicate that prion aggregates traffic via mature autophagosomes or autolysosomes.

### Spermine treatment clears prion aggregates in SMB cells and CAD cells terminally differentiated into neurons and reduce ROS production in CAD cells

**Figure 2:**
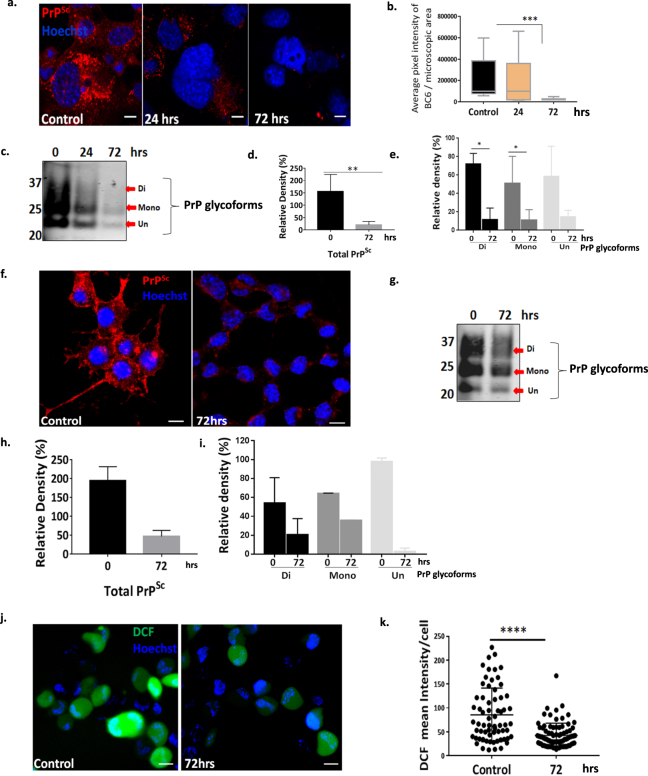
Spermine treatment clears prion aggregates in SMB and CAD cells terminally differentiated into neurons and reduces ROS in CAD cells. SMB.s15 and CAD22L cells were treated with 5 μM spermine for the mentioned period. (**a**) Representative immunofluorescence images showing reduced PrP^Sc^ staining after 24 and 72 hrs of spermine treatment in SMB.s15 cells (N = 3). (**b**) Significantly reduced pixel intensity of PrP^Sc^ in immunofluorescence images on 72 hrs spermine treatment in SMB.s15 cells. (**c**) Proteinase-K treated lysates immunoblotted for PrP^Sc^ after 0, 24 and 72 hrs of treatment with spermine in SMB.s15 cells (N = 3). (**d**) Significantly reduced relative density of total PrP^Sc^ after 72 hrs of spermine treatment. (**e**) Significantly reduced relative density of di- and mono- glycosylated forms of PrP^Sc^ after 72 hrs of spermine treatment. (**f**) CAD22L cells showing reduced immunofluorescence for PrP^Sc^ after 72 hrs of spermine treatment (N = 2). (**g**–**i**) Reduced expression of total PrP^Sc^ and PrP^Sc^ glycoforms in CAD22L cells, after 72 hrs of spermine treatment (N = 2). PrP^Sc^ glycoforms are shown with red arrows. (**j**) Reduced DCF fluorescence in CAD22L cells after 72 hrs of spermine treatment (N = 2). (**k**) Significantly reduced DCF mean intensity in CAD22L cells. Scale bar = 10 μm. Respective full length blots are shown in Supplementary Fig. 3. Abbreviations used: Chloroquine (CQ), Transmission Electron Microscopy (TEM), Dichlorofluorescein (DCF).

Given that spermidine protects against de novo formation of yeast prions^18^ and role of dextran-spermine in PrP^Sc^ clearance in chronically infected N2a-M cells^19^, we investigated if spermine has a similar effect in SMB and CAD22L cells (CNS catecholaminergic cells infected with the prion agent 22L)^26^. We performed an initial investigation using both spermidine and spermine towards clearance of PrP^Sc^ aggregates. The dosage which was found to be equally effective both in spermidine and spermine were compared using immunoblotting after 72 hrs of treatment (Supplementary Fig. 1a). Spermine was consistently found more effective compared to spermidine; hence we decided to further investigate spermine only. We primarily determined the correct dosage of spermine; at 20–100 μM extensive cell death was observed and at the lower concentrations (1–4 μM) the cells remained viable but no clearance of PrP^Sc^ was observed. However, treatment with an intermediate dose of 5 μM of spermine was found to be consistently effective in clearing prion aggregates and the cells survived as untreated cells. Dose dependent cell viability was assessed using MTT assay (Supplementary Fig. 2a1). We then stained the cells with BC6 and observed a significant reduction in the intensity of PrP^Sc^ in a time dependent manner (Fig. 2a,b). A significant reduction in total PrP^Sc^ was also observed on WBs of cell extracts (Fig. 2c,d). In particular, a significant reduction in both the di- and mono- glycosylated forms were observed after 72 hrs of treatment (Fig. 2c,e), confirming that 5 μM spermine is able to effectively reduce the levels of PrP^Sc^ in SMB.s15 cells. We further applied spermine treatment in CAD22L cells that were terminally differentiated into neurons and observed similar results as seen in SMB.s15 cells after 72 hrs of treatment with 5 μM spermine, both by microscopy (Fig. 2f) and WBs (Fig. 2g–i) with the BC6 antibody. Oxidative stress is a known inducer of neuronal cell death associated with prion diseases^27^ and spermine is a known scavenger of ROS^22^. We used TMRE (tetramethylrhodamine, ethyl ester) which assesses changes in mitochondrial membrane potential (Δψm) and 2′,7′-dichlorofluorescin diacetate (DCFDA) assay to assess the levels of 2′,7′-dichlorofluorescein (DCF) fluorescence. Both of these assays are indicators of ROS. It has been previously demonstrated that mitochondria with increased membrane potential produce more ROS^28^. We observed a significant decline in mitochondrial membrane potential (Supplementary Fig. 2a2) along with a significant decline in ROS levels after 72 hrs of spermine treatment (Fig. 2j,k) in CAD 22L cells. Thus, spermine treatment is not only able to clear PrP^Sc^ aggregates but also reduces the levels of ROS in prion infected cells.

### Spermine clears prion aggregates by enhancing autolysosomal flux

**Figure 3:**
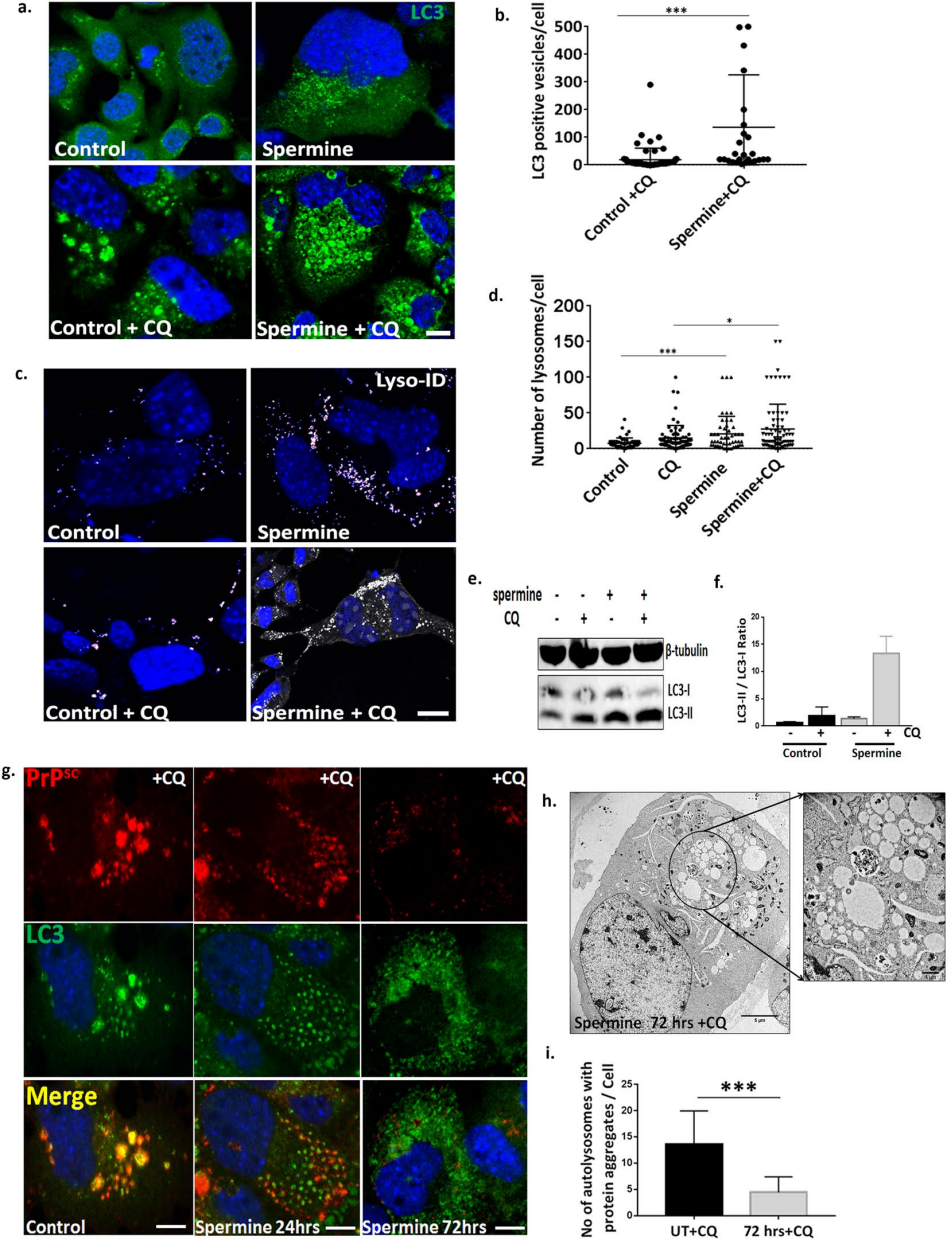
Spermine clears prion aggregates by enhancing autolysosomal flux. SMB.s15 cells were either left untreated or treated with 10 μM CQ or treated with 5 μM Spermine alone or 5 μM Spermine and 10 μM of CQ for 24 hrs. (**a**) Representative images showing increased number of LC3 positive vesicles on spermine treatment (N = 3). (**b**) Significantly increased LC3 positive vesicles on spermine + CQ treatment compared to control + CQ treatment. (**c**) Representative images of increased number of Lyso-ID positive lysosomes on spermine treatment (N = 3). (**d**) Significantly increased lysosomes on spermine treatment. (**e**) Representative immunoblot to show increased conversion of LC3I to LC3II on spermine and CQ treatment (lanes 3, 4) compared to untreated cells (Lanes 1, 2) (N = 3). (**f**) Quantification of LC3II/ LC3I ratio as a marker of autophagic flux. SMB.s15 cells were treated with 5 μM spermine for 0, 24 and 72 hrs and 10 μM of CQ was given 16 hrs before the end point of spermine treatment to visualise the autolysosomes. (**g**) Representative immunofluorescence images of SMB.s15 cells stained for PrP^Sc^ and LC3 antibodies (N = 3). (**h**) Representative TEM image after 72  hrs of spermine treatment showing vesicles with reduced aggregates, area inside the circle is enlarged to show the vesicles (N = 2). (**i**) Vesicles with significantly reduced PrP^Sc^ aggregates on 72 hrs of spermine treatment. Scale bar for confocal images = 10 μm. Respective full length blots are shown in Supplementary Fig. 3. Abbreviations used: Chloroquine (CQ), Transmission Electron Microscopy (TEM).

Spermine is known to induce autophagy in the human fibrosarcoma cell line HT1080^21^. We therefore investigated if spermine has a similar effect on the autophagic flux in SMB.s15 cells. Cells were treated both with the 5 μM spermine and 10 μM CQ for 24 hrs. A significant increase in the number of LC3 positive vesicles was observed after 24 hrs of spermine treatment (Fig. 3a,b). It is important to note that the morphology of LC3 punctae showed distinct changes when CQ was added into the media to stop the autophagic flux. The LC3 punctae were observed to be bigger in size and localised to circular vesicle-like structures upon CQ treatment compared to no treatment or treatment with spermine where numerous tiny LC3 punctae were observed. We also observed a significant increase in lysosomes using Lyso-ID a live dye for staining lysosomes (Fig. 3c,d). After 24 hrs spermine treatment we observed an increased conversion of LC3-I to LC3-II by WB with LC3 antibody compared to untreated cells with and without CQ treatment demonstrating an increased autophagic flux (Fig. 3e,f). In summary spermine treatment is not only able to enhance the number of autophagosomes but it is also making more lysosomes available for degradation.

We further investigated the levels of PrP^Sc^ aggregates within autophagosomes/autolysosomes after 0, 24 and 72 hrs of spermine treatment and 16 hrs CQ in the SMB.s15 cells. The cells were stained with BC6 and LC3 and observed under confocal microscope. After 24 hrs of treatment (Fig. 3g second column) there were fewer PrP^Sc^ aggregates in the LC3 marked autolysosomes when compared to 0hrs i.e. untreated SMB.s15 cells (Fig. 3g first column) and by the end of 72 hrs there were minimal LC3 positive autolysosomes or PrP^Sc^ aggregates remaining (Fig. 3g last column) indicating that spermine’s action has effectively cleared the aggregates within these cells. This result further confirms the reduced levels of PrP^Sc^ seen on spermine treatment in SMB.s15 and CAD cells (Fig. 2a,b,f). After 72 hrs of treatment many cells showed empty vesicles by TEM (Fig. 3h,i), compared to untreated SMB.s15 cells full of vesicles with aggregates (Fig. 1d, SMB.s15), these are single membrane vesicles likely to be autolysosomes which have digested the PrP^Sc^ aggregates and thus cleared of any protein aggregates (Fig. 3g,h).

### Blocking formation of active autophagosomes with PI3K inhibitor LY294002 and silencing essential autophagy gene *Atg5* prevents cells from clearing prion aggregates

**Figure 4:**
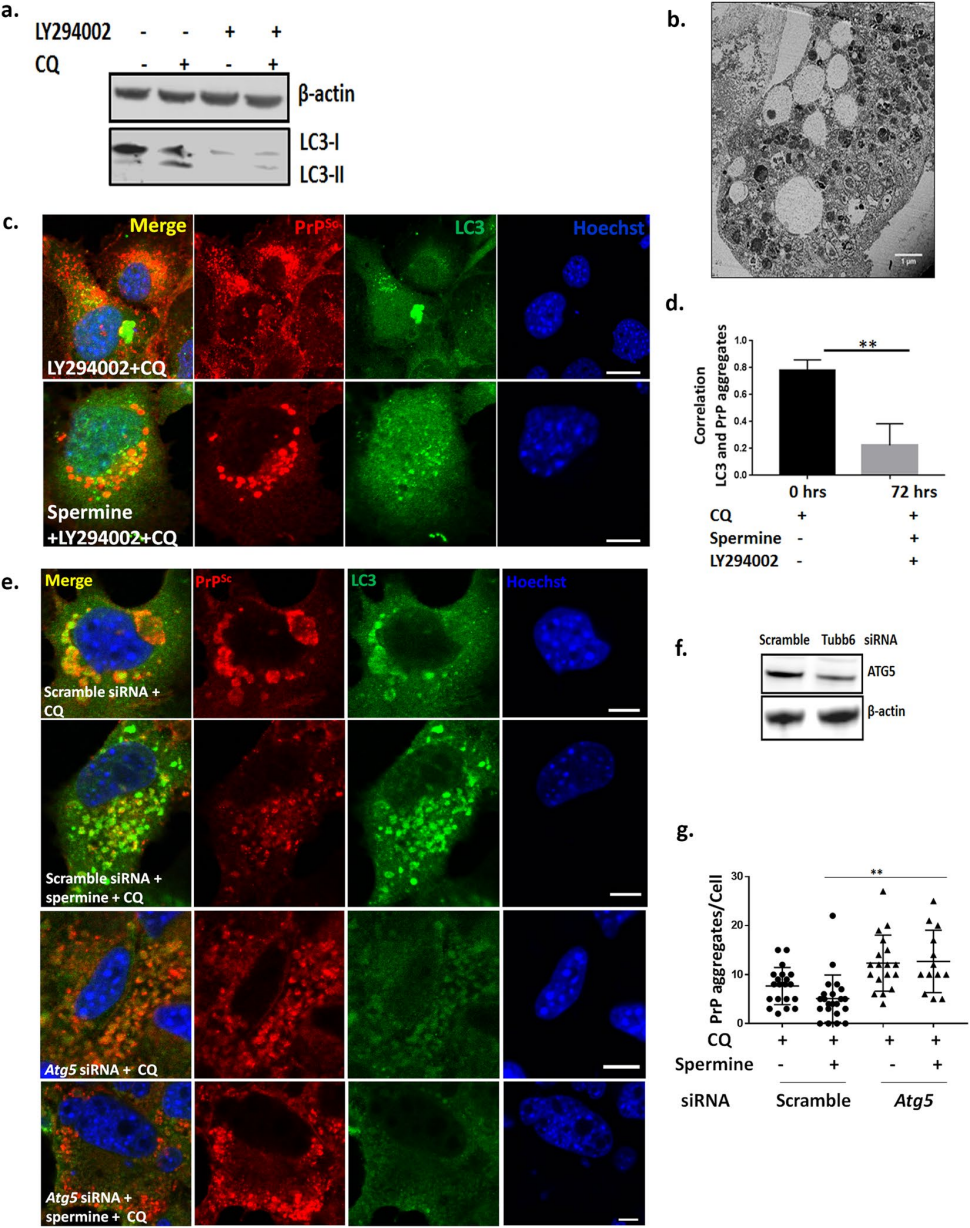
Blocking formation of active autophagosomes with PI3K inhibitor LY294002 and *Atg5* siRNA leads to failure of clearance of prion aggregates. SMB.s15 cells were treated with 40 μM PI3K inhibitor LY294002 for 16 hrs to block the formation of autophagosomes. (**a**) Representative immunoblot to show reduced LC3-I and LC3-II after LY294002 treatment, lane 3 and 4. (**b**) Representative TEM image showing aggregates scattered in the cytoplasm with empty vesicles after treatment with 5 μM spermine for 72 hrs, LY294002 and 10 μM CQ for the last 16 hrs. (**c**). Representative immunofluorescence images showing reduced colocalisation of LC3 with PrP^Sc^ aggregates after treatment with 5 μM spermine for 72 hrs, LY294002 and 10 μM CQ for the last 16 hrs. (**d**) Significantly reduced colocalisation between LC3 and PrP^Sc^ aggregates on LY294002 treatment. For the *Atg5* siRNA treatment, the cells with either treated with 10 μM of CQ or 5 μM spermine and10μM of CQ for 24 hrs post transfection. (**e**) Representative immunofluorescence images showing reduced LC3 punctae and increased prion aggregates on treatment with *Atg5* siRNA when compared to scrambled control siRNA. Treatment with spermine does not reduce the number of aggregates after *Atg5* siRNA treatment (last lane). (**f**) Western blot to show reduced *Atg5* expression on treatment with *Atg5* siRNA when compared to scrambled control siRNA and the loading control β- actin. (**g**) Graph showing a significant increase in the number of PrP aggregates on spermine treatment post *Atg5* siRNA treatment compared to spermine treated cells from scramble control siRNA. N = 2, Scale bar = 10 μm. Respective full length blots are shown in Supplementary Fig. 3. Abbreviations used: Chloroquine (CQ), Transmission Electron Microscopy (TEM).

LY294002 (2-(4-morpholinyl)-8-phenylchromone) is a potent, cell permeable inhibitor of phosphatidylinositol 3-kinase (PI3K) and a known inhibitor of autophagosome formation^29^. Treatment of SMB.s15 cells with 40 μM of LY294002, with and without 10 μM of CQ reduced both LC3-I and its lipidated form LC3-II (Fig. 4a). When the cells were treated with 40 μM of LY294002 in the presence of 5 μM spermine for 24 and 72 hrs, no difference in PrP^Sc^ accumulation was detected compared to untreated samples (data not shown). However, when the cells treated with 5 μM spermine for 72 hrs were given 40 μM LY294002 and 10 μM CQ together for 16 hrs to assess the accumulation of PrP^Sc^ aggregates and stained with LC3 and BC6, increased accumulation of PrP^Sc^ aggregates was observed in the cytoplasm (Fig. 4b). Furthermore, upon LY294002 treatment we observed a significant reduction in the colocalisation of LC3 positive punctae to the PrP^Sc^ aggregates (Fig. 4c) compared to SMB.s15 cells treated with 10 μM CQ only (Fig. 1i, CQ treatment and Fig. 3g first column) where the PrP^Sc^ aggregates were seen surrounded by LC3 positive vesicles with a high degree of co-localization. A significant decline in correlation between LC3 punctae and PrP^Sc^ aggregates was observed upon LY294002 treatment (Fig. 4d). Moreover, TEM of the cells treated with 5 μM spermine for 72 hrs and given 40 μM LY294002 and 10 μM CQ for 16 hrs clearly show aggregates lying in the cytoplasm around empty vesicles (Fig. 4b). Again these images are clearly distinct from the CQ treated SMB.s15 cells, in which all the PrP^Sc^ aggregates were observed inside vesicles (Fig. 1d SMB.s15) or very few aggregates seen in the autolysosomes on spermine treatment (Fig. 3h). We further silenced the essential autophagy gene *Atg5* using siRNA. *Atg5* is required for the formation of active autophagosomes^30^. Silencing of *Atg5* in SMB.s15 cells resulted in reduced *Atg5* expression (Fig. 4f) and complete loss of LC3 punctae (Fig. 4e, the last two rows). Interestingly silencing of *Atg5* resulted in a significant increase in PrP^Sc^ aggregates which could not be rescued by spermine treatment (Fig. 4e,g). These results clearly indicate that aggregate clearance by spermine is autophagy dependent and no engulfment of PrP^Sc^ aggregates takes place in the cytoplasm when autophagy is inhibited.

### Spermine increases the acetylation of proteins involved in retrograde transport

**Figure 5:**
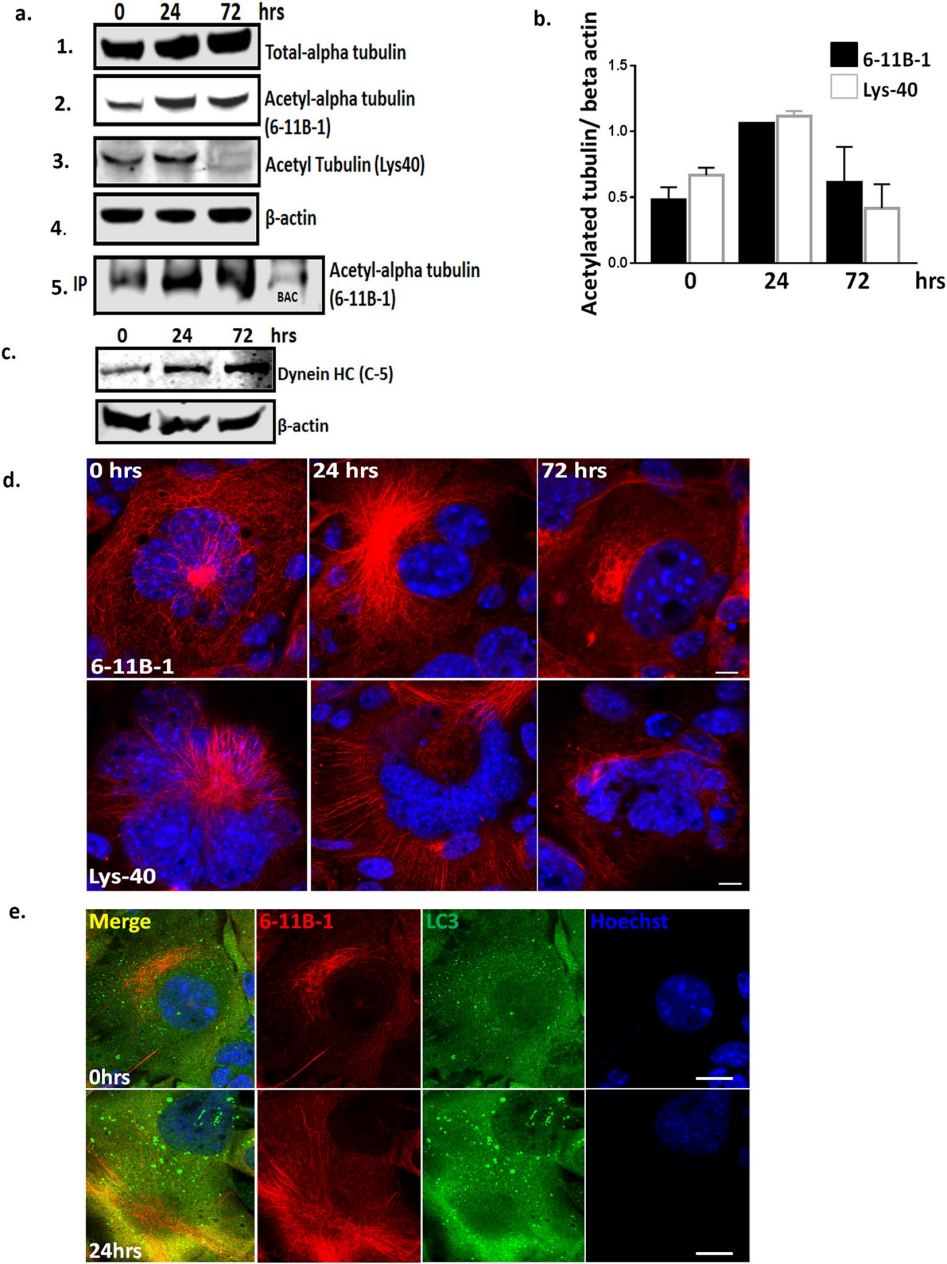
Spermine increases the acetylation of proteins involved in retrograde transport. SMB.s15 cells were treated with 5 μM Spermine for 0, 24 and 72 hrs; cytoplasmic extracts were used both for immunoprecipitation (IP) with anti-acetyl lysine antibody and run on WB or run directly on WB. (**a**) Immunoblotting with total alpha tubulin (lane1), two different acetyl-alpha tubulin antibodies (6–11B-1 and Lys40) show increased expression (lane 2 and 3) after 24 hrs spermine treatment, and loading control β-actin (lane 4) both from the cytoplasmic extract as well as from the immunoprecipitation product after IP with acetyl-lysine antibody (lane 5), BAC (lane 5 last row) (**b**) Graph showing relative expression levels of 6-11B-1 and Lys40 compared to β-actin. (**c**) WB shows increased expression of approx. 500 kDa Dynein-HC after 24 and 72 hrs of spermine treatment (**d**) Representative images with acetyl-alpha antibodies (6-11B-1, first lane) (Lys40, second lane) show increased expression on 24 hrs spermine treatment. (**e**) Representative images with increased colocalisation of bright LC3 punctae with acetyl-alpha antibody (6-11B-1) after 24 hrs spermine treatment. Scale bar = 10 μm, N = 3 for LC-MS, N = 2 for WB and immunofluorescence. Respective full length blots are shown in Supplementary Fig. 3. Abbreviations used: Immunoprecipitation (IP), Western blot (WB), Beads Alone Control (BAC), Liquid chromatography–mass spectrometry (LC-MS).

Acetylation of proteins is known to control the process of autophagy^31^. We therefore used an affinity purification mass spectrometry (MS) approach in order to examine the acetylation of proteins and thus determine spermine’s mechanism of action. Cytoplasmic lysates from SMB.s15 cells were pulled down with an acetylated lysine antibody after 0, 24 and 72 hrs of treatment with spermine. These pull downs were trypsin digested and analysed by nanoLC-MS. We observed a significantly greater number of potentially acetylated proteins identified by MS after 24 hrs spermine treatment compared to 72 hrs, untreated, and beads alone control. Staining with InstantBlue protein stain showed a similar increase at 24 hrs on the gels compared to total protein blots (Supplementary Fig. 1b). The proteins with high mascot scores and increased number of peptide matches after 24 hrs treatment were cytoskeletal tubulins (Tubulin alpha1B/1C, Tubulin beta-5 chain, Tubulin beta-4B) (Supplementary Fig. 1c). These high scores and increased peptide matches were consistent for all three MS runs. This suggests these proteins are either increased in expression or acetylated by spermine treatment. To further verify the MS results two different antibodies were used against acetylated alpha tubulins (6-11B-1 and Lys40) and assessed both by WB and immunofluorescence. An up regulation in both total and acetylated alpha tubulins was observed following 24 hrs of spermine treatment (Fig. 5a lane 1, 2 & 3) on WBs when compared to β-actin expression (Fig. 5a lane 4, 5b). A similar result was also seen on the acetylated-lysine pull down product, supported by the observation that these proteins were reduced both in untreated and the beads alone control (BAC) (Fig. 5a lane 5) supporting our observation from the LC-MS data (Supplementary Fig. 1b). Further confocal imaging of SMB.s15 cells treated with 5 μM spermine for 24 and 72 hrs was carried out after staining with both of the acetylated alpha tubulin antibodies. An increase in acetylated alpha tubulins was observed at 24 hrs (Fig. 5d) and additionally an increased colocalization of LC3 punctae on acetylated alpha tubulins was apparent after 24 hrs of spermine treatment compared to untreated cells (Fig. 5e). Furthermore, MS results from anti-acetyl lysine pull downs also identified dynein-HC (Supplementary Fig. 1b) after 24 hrs of spermine treatment. However, we were unable to determine the acetylation status of the dynein-HC as there is no acetylated dynein antibody available. It may well not be acetylated itself, rather identified here as it has strong affinity for tubulins^32^. To identify the most likely explanation the expression of total dynein-HC in the cytoplasmic extract was assessed on spermine treatment and an increase in its expression both at 24 and 72 hrs post spermine treatment was observed (Fig. 5c). These results strongly suggest an increased acetylation of cytoskeletal proteins involved in retrograde transport of autophagosomes on spermine treatment.

### Microtubule protein Tubb6 binds to prion aggregates and targets them to autophagosomes

**Figure 6:**
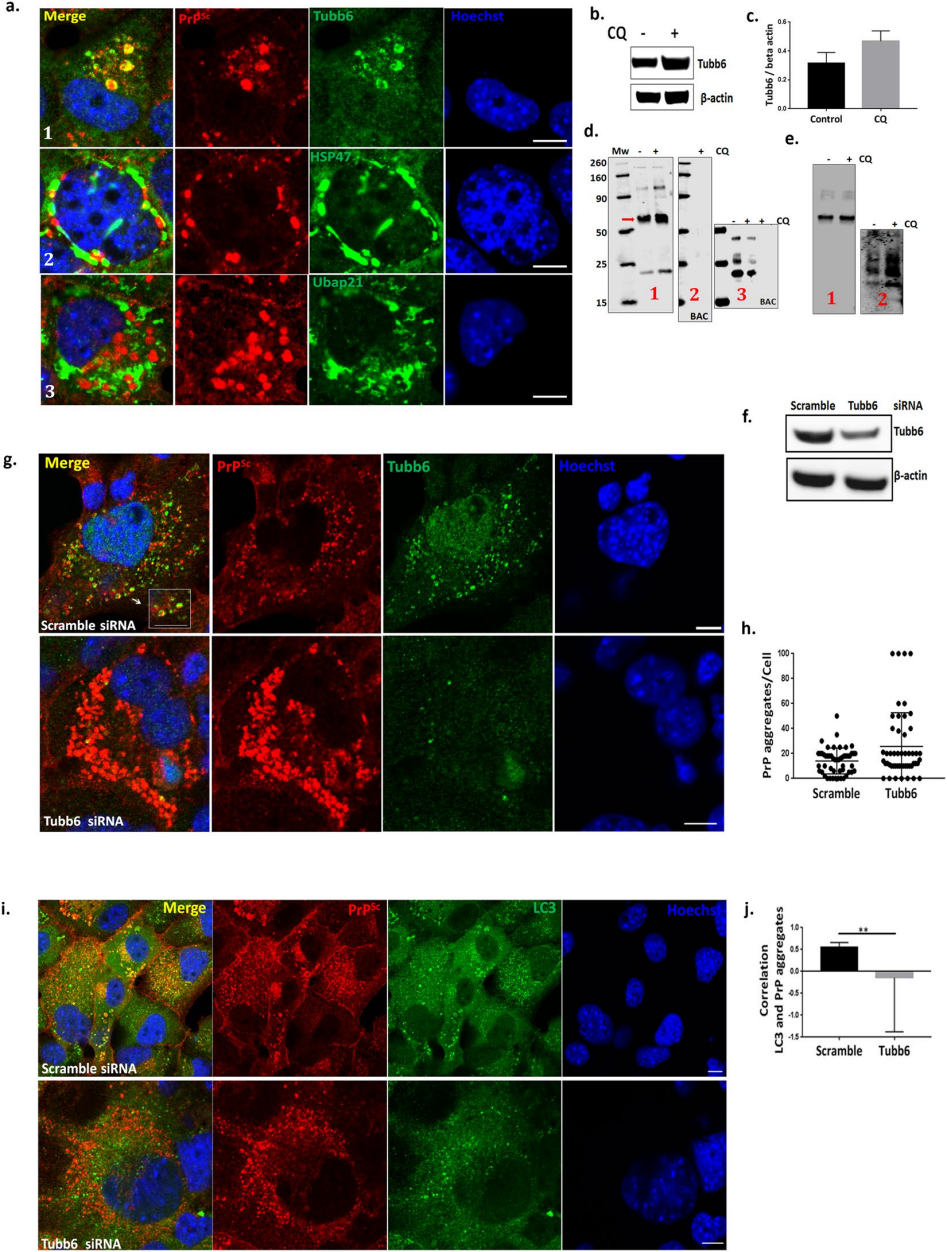
Microtubule protein Tubb6 binds to prion aggregates and targets them to autophagosomes. All the experiments were performed on SMB.s15 cells with or without 10 μM CQ for 16 hrs. (**a**) Representative immunofluorescence images of Tubb6, lane 1 (N = 3), HSP47, lane 2 (N = 2) and Ubap21, lane 3 (N = 2) with PrP^Sc^ showing localisation of individual protein targets with respect to PrP^Sc^ in the cytoplasm. (**b**) Representative WB show increased expression of Tubb6 on CQ treatment (N = 2). (**c**) Relative expression levels of Tubb6 compared to β-actin on CQ treatment. (**d**) (1) IP with BC6 (anti-PrP antibody) immunoblotted with Tubb6, red arrow indicates approx. 53 kDa Tubb6 band (2) Beads alone control (3) immunoblotted for BC6 after PK treatment. (**e**) Reverse IP with Tubb6 (1) immunoblotted with Tubb6 (2) immunoblotted for BC6 after PK treatment. (**f**) Representative WB to show reduced expression of Tubb6 on siRNA treatment. (**g**) Representative immunofluorescence images of increased number of PrP^Sc^ aggregates after Tubb6 siRNA treatment compared to scramble siRNA, magnified area inside the white square to show PrP^Sc^ aggregates are surrounded by Tubb6 in scramble siRNA control (N = 3). (**h**) Increased numbers of PrP^Sc^ aggregates on Tubb6 siRNA treatment (non-significant P = 0.0668). (**i**) Representative immunofluorescence staining for LC3 and BC6 after 10 μM CQ treatment showing no targeting of PrP^Sc^ aggregates to LC3 positive punctae after Tubb6 siRNA treatment compared to the control cells treated with scramble siRNA (N = 3). (**j**) A significant negative correlation between LC3 and PrP^Sc^ aggregates after Tubb6 siRNA treatment compared to the control cells treated with scramble siRNA. Scale bar = 10 μm. Respective full length blots are shown in Supplementary Fig. 3. Abbreviations used Chloroquine (CQ), Proteinase K (PK), Immunoprecipitation (IP).

Using affinity-purification methods coupled with MS we investigated whether there are any specific proteins which bind to PrP^Sc^ aggregates and chaperone them to autophagosomes. Immunoprecipitation (IP) was carried out with prion specific antibody (BC6) in SMB.s15 cell lysates with and without CQ treatment. Simultaneous pull downs were carried out with lysates including beads without antibody to check for non-specific binding. On-bead protease digestion was carried out before MS runs. While a number of protein hits were obtained from each pull down only three proteins (Tubb6, Hsp47 and Ubap21) were selected as potential interactors based on their presence in the CQ treated samples, reduced levels as reflected by mascot scores and the number of peptides or the absence from untreated samples and absence from pull downs using beads alone control (without antibody) (Supplementary Fig. 2). In order to establish the localisation of these target proteins with respect to PrP^Sc^ aggregates, SMB.s15 cells were co-stained with antibodies against these proteins and BC6 after CQ treatment. Confocal images clearly showed the colocalisation of Tubb6 in circular vesicles with PrP^Sc^ aggregates (Fig. 6a row 1) whereas the other two targets, HSP47 and Ubap21 did not show similar colocalisation (Fig. 6a row 2, 3). We also observed a trend towards increased expression of Tubb6 after CQ treatment in WB (Fig. 6b,c). We further validated Tubb6 for its binding efficiency with PK resistant PrP. The resultant BC6 pull down product and BAC was run on WBs and immunoblotted for Tubb6 (Fig. 6d, 1, 2), and PK digested bead product was immunoblotted for PrP^Sc^ (Fig. 6d, 3). Both Tubb6 and PrP^Sc^ were found in the IP products, demonstrating Tubb6 does bind to PrP^Sc^. In order to confirm this binding, we reversed the IP and immunoprecipitated with Tubb6 and observed the expression of both the Tubb6 and PrP^Sc^ in the IP product (Fig. 6e 1 & 2).

We further asked whether Tubb6 has a more specific role in targeting PrP^Sc^ aggregates to the autophagosomes using RNAi on SMB.s15 cells (Fig. 6f). Our experiments show an increase in PrP^Sc^ aggregates after Tubb6 siRNA treatment compared to scramble siRNA control (Fig. 6g,h). These aggregates are not targeted to autophagosomes as shown in Fig. 6i lower panel where these protein aggregates can be seen around the bright punctate LC3 compared to scramble siRNA treated control cells where protein aggregates can be seen localised with LC3 punctae (Fig. 6i upper panel). A significant negative correlation was observed between LC3 and BC6 on Tubb6 siRNA treatment when compared to the scrambled siRNA treated control (Fig. 6j).

### Spermine enhances colocalisation of Tubb6 with PrP^Sc^ aggregates in aggresome like bodies

**Figure 7:**
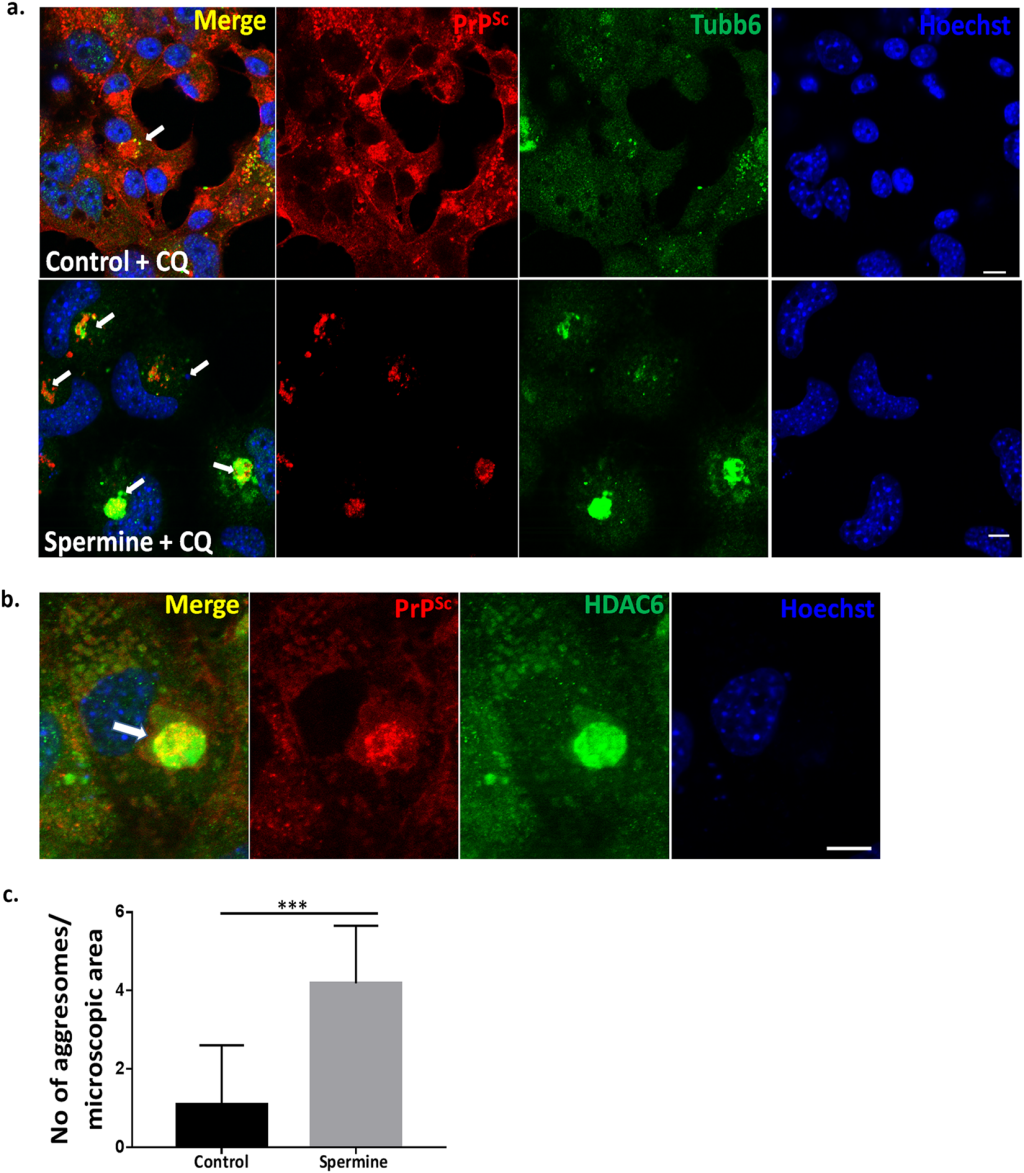
Spermine enhances colocalisation of Tubb6 with PrP^Sc^ aggregates in aggresomes. SMB.s15 cells were treated with10 μM CQ or treated with 5 μM of spermine and 10 μM CQ for 24 hrs. (**a**) Representative immunofluorescence images of spermine + CQ treated cells showing cells with aggresomes stained with antibodies for Tubb6 and PrP^Sc^ aggregates (lower lane) compared to untreated control cells + CQ (upper lane) (N = 3). (**b**) Representative immunofluorescence image showing colocalisation of HDAC6 and PrP^Sc^ aggregates (N = 3). (**c**) Significantly increased number of cells per microscopic area showing aggresomes on spermine + CQ treatment when compared to untreated control cells+ CQ, each microscopic area contained 5–15 cells, total of 10 microscopic areas were counted. Scale bar = 10 μm. Abbreviations used Chloroquine (CQ).

Aggresome formation is a process to bring potentially cytotoxic aggregates into one location for their eventual autophagic clearance from the cell^33^. After 24 hrs of spermine and CQ treatment we observed single aggresomes (Fig. 7a, spermine) which were bigger in size compared to PrP^Sc^ aggregates seen in SMB.s15 cells (Fig. 1i, CQ treated) and also fewer cells showed them without spermine treatment (Fig. 7a control, 7c). These aggresomes stained positive for both BC6 and Tubb6 (Fig. 7a) and BC6 and HDAC6 (an aggresome marker)^34^, confirming that they are indeed aggresomes (Fig. 7b). It is well established that aggresome formation is a cytoprotective response to sequester potentially toxic misfolded proteins and facilitate their clearance by autophagy^35^. Thus, our data demonstrates that spermine treatment is able to facilitate formation of these aggresomes in which Tubb6 is seen colocalising to PrP^Sc^ aggregates.

## Discussion

In this study we have demonstrated that the natural polyamine spermine can clear the prion aggregates in prion infected cell cultures. We also show that spermine treatment can significantly reduce the associated ROS in neuronal cells infected with the prion agent 22L. This aggregate clearance is dependent on the cellular autophagy process. Our affinity-MS experiments provide strong evidence of an increase in acetylated tubulins known to have role in stabilising microtubules and facilitating cellular retrograde transport of autophagosomes. Using a similar approach, we have also identified microtubule protein Tubb6 as a binding partner of PrP^Sc^ aggregates which targets them to autophagosomes.

Various polyamine based strategies have been tested for degradation of detergent-insoluble and PK-resistant PrP^Sc^. Exogenous administration of polycationic DOSPA bound to liposomes was found to significantly reduce the levels of PrP^Sc^ in ScN2a cells^36^. PPI Poly(propylene imine) dendimers, a group of branched polyamines have been implicated in denaturation of PrP^Sc^ and its clearance, this is shown to be occurring inside lysosomes^37,38^. However, in the absence of autophagy markers the role of the autophagy pathway during the clearance of PrP^Sc^ in these studies could not be assessed. Furthermore, polyamine spermidine has been shown to successfully ameliorate neurodegeneration in various *in vitro*, mouse and fly model of diseases including ischaemia^39^, normal tension glaucoma^40^, proteinopathies of the TAR DNA-binding protein 43^41^ Parkinson’s disease^42^ and in a yeast model of prion diseases^43^. However, its derivative spermine has not yet been investigated for its therapeutic effects.

A study by Bera and Nandi in 2007 reported spermine as a more potent polyamine than spermidine in inhibiting nucleic-acid-induced polymerisation of prion protein^20^ and its role as a free radical scavenger has also been shown^22^. Spermine is a mimetic of caloric restriction (CR) and the role of caloric restriction in enhancing longevity is well known. However its role in degradation of protein aggregates has not been addressed in mammalian systems^44^. CR mimetics like spermidine are known to enhance longevity via enhancing autophagy^45^, this could be supported with the observation that high levels of spermine were measured in the whole blood of healthy nona/centenarians^46^. Thus, both spermine and spermidine may have a role in clearance of protein aggregates seen during neurodegeneration associated with aging.

We and others have reported impaired autophagy pathway in aging cells^47,48^ and it has been shown that the aging brain can trigger the formation of amyloid aggregates though the mechanisms remain unknown^49^. Massive accumulation of autophagic vacuoles within large swellings along dystrophic and degenerating neurites is common in AD and other protein misfolding diseases and this could be related to defects in various steps of the autophagy pathway such as initiation of autophagosome formation^50,51^, cargo recognition^52^, autophagosome-lysosome fusion^53,54^, lysosomal proteolysis^55,56^ as well as a defect in retrograde transport responsible for the trafficking of cargo filled autophagosomes from the cellular periphery to the lysosomes residing near the nuclear core^57^. While compounds enhancing autophagy are being tested in various *in vitro* and *in vivo* models of neurodegeneration^8,14,41,43,58^ their clinical success is still to be proven. Our experiments with the natural polyamine spermine reveal not only an increase in the number of active autophagosomes but also in number of lysosomes. This two way enhancement (Fig. 3a,c) provides the cells with an opportunity to sequestrate more of the misfolded protein aggregates and also provide access to increased number of lysosomes with proteases for their degradation. As the PrP^Sc^ is likely to be trafficked via endosomes^59^ from the cellular surface and passed over to autophagosomes to be delivered to lysosomes, an efficient retrograde transport system consisting of stable microtubules is a key to successful delivery of cargo filled autophagosomes to nuclear periphery where the bulk of lysosomes assemble.

Microtubular proteins often function in complexes and are heavily regulated at the post-translational levels. Acetylation levels of many of these proteins may provide a pro-autophagic response and this has been shown for compounds like spermidine and resveratrol^60^. Acetylated microtubules are required for the fusion of autophagosomes with lysosomes to form autolysosomes^61^ and to facilitate phagophore/autophagosome formation by specifically recruiting BECN1, class III PtdIns3K, WIPI1, ATG12–ATG5 and LC3-II on unstable microtubules^62,63^ and also to facilitate the movement of mature autophagosomes (marked with LC3-II) along stable microtubules^64^. Lys40 acetylation marks stable microtubules and it has been shown that α-tubulin acetylation at Lys40 increases the recruitment and mobility of KIF1 and dynein both *in vitro* and *in vivo*^65^. Dynein-mediated autophagosome trafficking has also been demonstrated to contribute to the formation of autolysosomes and thus enhance the autophagic flux^66^. The role of dynein and tubulins become more critical in neuronal cells as they are employed for the retrograde transport of aggregated proteins from the neurites and synaptic terminals to mature acidic lysosomes which are relatively enriched in the soma. Deficiency of these could result in accumulation of autophagic vesicles in these regions. It has been reported that spermidine promotes acetylation of histones and thus regulate the expression of autophagy genes (17); however the effect of these polyamines on cytoskeletal proteins has yet to be investigated. We speculate that spermine may act in a similar fashion. We hypothesise that spermine treatment is able to facilitate retrograde transport by hyperacetylating tubulins and increasing the expression of Dynein-HC.

Along with efficient transportation, selective targeting of the cargo for autophagic degradation is known to increase the efficiency of aggregate degradation^67^. The presence of γ-tubulin in aggresomes is reported in few studies^68,69^. Using the affinity purification-MS approach we have shown for the first time that Tubb6 can be seen co-localised to prion aggresomes. Additionally, silencing of Tubb6 results in an increased number of prion aggregates and mis-targeting of prion aggregates to the LC3 positive autophagosomes, thus establishing its quintessential role in tagging PrP^Sc^ aggregates for degradation via autophagy. Our online search at iLIR database^70^ shows the presence of at least 4 LIR (WxxL) motifs in the Tubb6 sequence with a PSSM score of 5, 7, 7 and 8 respectively, indicating the presence of motifs necessary for interaction with Atg8 (LC3)-family of proteins indicating a possibility of Tubb6 being an autophagy adaptor for prion aggregates, however more experiments will be needed to establish its bona fide role as an autophagy adaptor. Moreover, aggresome formation is a major cytoprotective mechanism formulated by a cell overwhelmed with protein aggregates, whereby cellular machinery attempts to centralize the collection of cargo filled autophagosomes to enhance the chances of their fusion with lysosomes and later degradation^33,71^. This seems to be aided by spermine along with acetylating microtubules which is known to facilitate transportation of autophagic vesicles. We also hypothesise that Tubb6 is a novel member of the aggresome-autophagy pathway which also includes HDAC6, dynein, acetylated tubulins and LC3.

**Figure 8:**
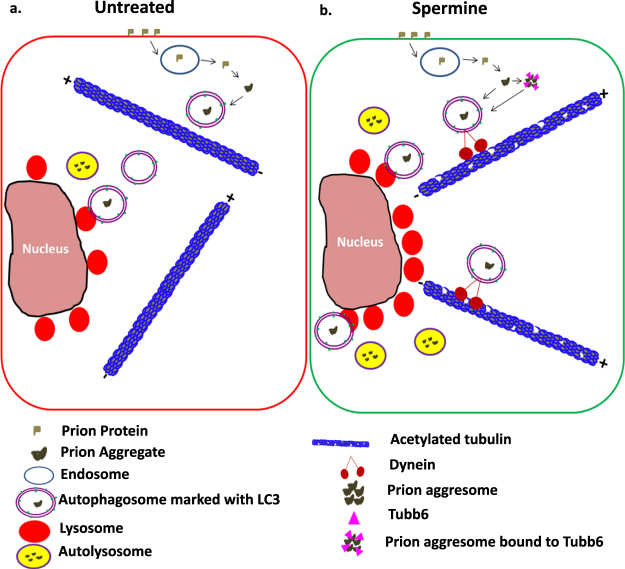
A pictorial summary of how spermine treatment facilitates degradation of prion aggregates in SMB.s15 cells. Spermine treatment clears prion aggregates by enhancing the autolysosomal flux both by enhancing the number of mature autophagosomes and lysosomes. Treatment with spermine enhances the expression of acetylated tubulins and increases the expression of dynein-HC. Both acetylated tubulins and dynein-HC are known to have a role in stabilising microtubules for retrograde transport of autophagosomes from the cellular periphery to the perinuclear location where the lysosomes reside. Furthermore, spermine treatment facilitates formation of prion aggresomes bound to Tubb6 for their delivery to autophagosomes for their clearance (**b**). Compared to the untreated control cells (**a**).

Our results clearly show that autophagy induction clears prion aggregates. Interestingly, recent work has demonstrated that mTOR inhibitor rapamycin strongly inhibits exosomal prion release which could have effects on lateral transfer of prions^72^. This action could be because of the enhanced flow of prion aggregates through autophagy pathway and their subsequent degradation which when blocked could lead to exosomal prion release. Although the present study does not provide any evidence of prion clearance by spermine treatment *in vivo*, our results in the *in vitro* systems clearly suggest spermine and the pathways modulated by it as a new therapeutic target of future research for protein misfolding diseases (Summary of the results, Fig. 8).

## Methodology

### Cell lines

#### SMB cells

This SMB.s15 cell line was established originally by culture from a brain taken from a mouse clinically affected by the Chandler scrapie isolate and is shown to be a cell line of mesodermal origin^23^. They were cultured 0.5–1.0 × 10^5^ cells/ml in complete DMEM (Invitrogen) medium supplemented with 10% FBS in 12 well plates and 1 in 10 dilution for growing in T75 culture flasks. These cell lines have PrP^Sc^ aggregates. The pentosan sulphate (PS) cured form of SMB.s15 cell line is called SMB-PS and is used as a control cell line as it lacks disease associated PrP^Sc^ aggregates.

#### CAD cells

These are mouse cells infected/uninfected with the prion strain 22L^73^. They were cultivated in Opti-MEM (Invitrogen) supplemented with 10% fetal calf serum (FCS). The cells were differentiated into neurons by cultivation in Dulbecco’s modified Eagle’s medium (DMEM)/F12 medium (BioWhittaker) supplemented with 0.5% FCS for 6 days^26^.

#### Antibodies

Following antibodies were used, mouse monoclonal ROS-BC6 (BC6) (anti-prion) 1.3 mg/ml (TSE Resource Centre, Cat no: RC 063^74^), rabbit polyclonal anti-LC3 (MBL, PM036) 1:3000 for WB and 1:300 for imaging, anti-acetyl alpha tubulin (6-11B-1) (Santa Cruz biotech, sc-23950) and mouse monoclonal anti- acetylated tubulin (Lys40) (Proteintech, cat no 66200-1-Ig) both used at 1:1000 for WB and 1:300 for imaging, mouse monoclonal anti-DyneinHC (C-5) (Santa Cruz biotech, sc-514579) 1:1000 for WB, rabbit polyclonal anti-Tubb6 (Aviva systems biology, ARP60382_P050) 1:1000 for WB and 1:300 for imaging, rabbit polyclonal anti-HSP47 (Abcam, ab77609), rabbit polyclonal anti-UbAP2L (Thermo scientific, PA5-29520) both 1:300 for imaging, rabbit monoclonal anti-HDAC6 (Abcam ab133493) used at 1:100 for imaging and rabbit polyclonal anti-beta actin (GeneTex, cat no GTX109639), Rabbit Polyclonal ATG5 (Sigma, A0731), used at 1:1000 for WB, secondary antibodies goat anti rabbit 488 (Thermo Fisher Scientific A11034) and goat anti mouse 647 (Thermo Fisher Scientific A21236) were both used at 1:500 for microscopy.

#### Spermine

Spermine (product code 18041) was bought from Cayman chemicals. 1M stock was prepared in distilled water and stored at −80 °C for long term usage. Final dilutions for experiments were made in the full DMEM media before adding into cultures.

#### Cellular lysates and protease digestion

The cells were washed twice with cold phosphate buffered saline (PBS), and whole-cell lysates were prepared at 4 °C in TL1 lysis buffer (50 mM Tris-HCl [pH 7.4], 0.5% Triton X-100, 0.5% sodium deoxycholate). Lysates were clarified by centrifugation at 1000 × g for 2 min, and the total protein concentration was estimated by microBCA assay (Pierce). To analyse the PrP^C^ content, 50 µg of protein was methanol precipitated, centrifuged at 22,000 × g for 20 min and pellets were dried. For PrP^Sc^, one hundred micrograms of protein in 200 µl TL1 buffer was digested at 37 °C for 2 hrs with 4 µg proteinase-K (PK) per 1 mg of protein as published previously^75^. PK digestion was stopped by addition of four volumes of absolute methanol. After overnight incubation at −20 °C, samples were further centrifuged at 22,000 × g for 30 min, and pellets were dissolved in 20 μl 2X Laemmli sample buffer and denatured by boiling at 100 °C for 5 min for SDS-PAGE analysis.

#### TEM

Cells post spermine or CQ treatment were lifted using Trypsin/EDTA, spun down and washed twice with PBS and fixed for 3 hrs at RT in 2.5% glutaradehyde in 4% PFA. Washed twice in PBS, finally suspended in PBS and handed over for sectioning. Samples were then post-fixed in 1% Osmium Tetroxide in 0.1 M Sodium Cacodylate for 45 min, and then washed in three 10 min changes of 0.1 M Sodium Cacodylate buffer. These samples were then dehydrated in 50%, 70%, 90% and 100% ethanol (X3) for 15 min each, then in two 10 min changes in Propylene Oxide. Samples were then embedded in TAAB 812 resin. Sections, 1 μm thick were cut on a Leica Ultracut ultramicrotome, stained with Toluidine Blue, and viewed in a light microscope to select suitable areas for investigation. Ultrathin sections, 60 nm thick were cut from selected areas, stained in Uranyl Acetate and Lead Citrate then viewed in a JEOL JEM-1400 Plus TEM. Representative images were collected on a GATAN OneView camera.

#### Confocal Microscopy

Zeiss LSM 710 inverted confocal microscope was used to obtain all the confocal microscopy images. SMB and CAD cells were grown on cover slips in 24 well plates (at a concentration of 4 × 106). In general cells were fixed for 20 min in ice cold 4% PFA at 4 °C. Fixed cells were washed and permeabilised with 0.1% triton X100 for 10 min at 4 °C and blocked with 2% FCS for 30 min at RT before staining with primary antibodies. To observe PrP^Sc^ aggregates an additional step of treating the cells with 5M guanidine isothiocynate for 5–10 min at room temperature after permeabilising them with 0.5% tritonX-100 in PBS was employed. Live imaging for lysosomes was done using LYSO-ID red dye (Enzo life sciences, ENZ-51005-0100) (1:1000) by incubating the cells for 30 min at 37 °C. Proteostat dye (Enzo life sciences, ENZ-51035) was used to visualise the aggregates, cells were washed and fixed and permeabilised as above and incubated with proteostat dye and Hoechst (1:100) and incubated in dark for 30 min at RT, and cells were later washed and mounted on glass slides.

#### MTT, TMRE and ROS Assay

Cell viability was measured using MTT (3-(4,5-dimethylthiazol-2-yl)-2,5-diphenyltetrazolium bromide) cell viability kit (Biotium, 3006) as described by manufacturers. In short 1 × 10^5^ cells were seeded in 96 well plate, cells were treated with different doses of spermine for 72 hrs. 10 μl of MTT solution was added to 100 μl medium in each well and incubated for 2 hrs at 37 °C. After 2  hrs, 200 μl of DMSO was added directly into the media. Absorbance was measured at 570 nm.

Mitochondrial membrane potential was measured using TMRE assay kit from (Abcam, ab113852) using manufactures instructions. In short 1 × 10^5^ cells were seeded in 96 well plate and treated with 5 μM of spermine for 72 hrs. Cells were then stained with 500 nM of TMRE for 20 min at 37 °C, washed twice with PBS and analysed at 549/575 Ex/Em using spectrophotometer (BioTeK Synergy HT).

Intracellular ROS was detected using Dihydrofluorescein diacetate (DCFH-DA) (Sigma, 292648), CAD cells were washed of the media and PBS was added and 5 μM of DCFH-DA was added to the cultures and cells were incubated at RT in dark for 30 min. The cells were washed 3X with PBS, mounted with prolong gold antifade reagent (Thermo fisher scientific, P36930) and visualised immediately using Zeiss LSM 710 inverted confocal microscope.

#### Immunoblotting

Protein was extracted using radioimmunoprecipitation assay buffer with supplemental Halt Protease Inhibitor Cocktail EDTA-free (Thermo scientific, 87785). Protein content was measured using Pierce™ BCA Protein Assay Kit (Thermo scientific, 23225) and samples boiled with 4X sample buffer and then run on a 4–12% Bis-Tris gel (Invitrogen) for PrP, LC3, tubb6 and acetylated tubulins. To resolve 500 kDa DyneinHC on gel, 3–8% Tris-acetate gels were used (Invitrogen). Proteins were transferred to PVDF membranes (Immobilon-FL transfer membrane, IPFL00010) and blocked overnight in LICOR blocking buffer. The membranes were incubated in primary antibodies (1: 3000) in LICOR blocking buffer, at 4 °C overnight. For all the blots β-actin or β-tubulin were run on the same gel except for the DyneinHC where β-actin was run separately. Secondary antibodies used were goat α-rabbit IRDye 680RD (926-68071) or goat α-mouse IRDye 680 RD (926-68070) (Licor, Cambridge, UK). Blot detection was carried out using Odyssey CLx Infrared Imaging System. The default setting used for blot image acquisition on Odyssey CLx Infrared Imaging system was resolution 169 μm, quality medium, laser intensities between 5–1.5.

#### Acetyl-Lysine Enrichment

SMB.s15 cells were treated with 5 μM of spermine for 0, 24 and 72 hrs. Cells were washed and cytoplasmic extracts were made using Nuclear/Cytosol fractionation Kit from BioVision (K266). Fifty microliters of Pierce protein A/G Magnetic beads (Thermo scientific, 88802) were added to the protein sample equivalent to 200 μg of protein followed by incubation on a rotator for 1 h at RT to remove any non-specific binding of protein to the beads. The sample was then magnetically separated and the supernatant transferred to a new tube. Ten microliters of rabbit monoclonal anti-acetyl-lysine antibody (Proteintech, 66200-1-Ig) was added and the mixture incubated on a rotator at 4 °C overnight. The next day, 100 μl of beads were added to the sample followed by incubation on a rotator for 1 hr at 4 °C. The beads were washed five times with 1 ml of IP lysis buffer and separated into two equal portions, one half was given a final wash with LC-MS grade water before on bead digestion and MS identification and analysis and the other half was eluted from the beads by adding 2X Laemmli sample buffer and boiling at 95 °C for 15 min. These samples were spun at 1500 rpm for 2 min and the supernatant harvested and run for WB. A control with beads alone and no antibody was used in all the runs.

#### BC6 pull down

To identify specific binders for PrP^Sc^, SMB.s15 cells were either treated with 10 μM CQ for 16 hrs or left untreated. 10 μM CQ was added to increase the likelihood of PrP^Sc^ being sequestered in autophagosomes/ autolysosomes hence increasing the likelihood of enriching the proteins binding to PrP^Sc^ being brought to autophagosomes or autolysosomes. The protein extracts were made with IP lysis buffer, the rest of the procedure was same as described for acetyl lysine pull down except that anti PrP antibody BC6 was used for the pull down.

#### RNAi treatment

SMB.s15 cell 70% confluent was used for all the experiments. Standard siRNA treatment was followed, in short 6 μl of both the Tubb6 and control SiRNA mouse (Santa Cruz biotech, sc-141449, sc-37007) were used for transfection. The cells were left to incubate for 7 hrs at 37 °C, after which the transfection media was removed and cells were left in full media for 48 hrs before making extracts for WB or fixing them for microscopy or spermine treatment. For *Atg5* siRNA studies 8 μl of both the *Atg5* and control SiRNA mouse (Santa Cruz biotech, sc-41446, sc-37007) were used for transfection.

#### ‘On-bead’ tryptic digestion and LC MS identification of proteins

Washed beads were resuspended in 10% Triflouroethanol (pH 8.0) with gentle vortexing for one hour and digested with sequencing grade modified trypsin (Promega), following reduction with 5 mM dithiothreitol and alkylation with 10 mM iodoacetamide. The resulting peptide mixture containing beads were spun at 800 × g and supernatant was collected. The digested peptides were cleaned up using Stagetips following a standard protocol^76^.

NanoflowLC-MS/MS was performed on a micrOTOF-II mass spectrometer (Bruker, Germany) coupled to an RSLCnano LC system (Thermo). Tryptic digest was delivered to a trap column (Acclaim PepMap100, 5 μm, 100 Å, 100 μm i.d. × 2 cm) at a flow rate of 20 μL/min in 100% solvent A (0.1% formic acid in LCMS grade water). After 4 min of loading and washing, peptides were transferred to an analytical column (Acclaim PepMap100, 3 μm, 100 Å, 75 μm i.d. × 25 cm) and separated at a flow rate of 300 nL/min using a 60 min gradient from 7% to 35% solvent B (solvent B, 0.1% formic acid in acetonitrile). The eluted peptides from LC were electrosprayed directly on to the mass spectrometer for MS and MS/MS analysis in a data-dependent mode of acquisition. The m/z values of tryptic peptides were measured using a MS scan (300–2000 m/z), followed by MS/MS scans of the six most intense ions. Rolling collision energy for fragmentation was selected based on the precursor ion mass and a dynamic exclusion was applied for 30 sec.

Raw spectral data were processed with DataAnalysis (Bruker) software and the resulting peak lists were searched using Mascot 2.4 server (Matrix Science, London, UK) against Uniprot Mouse sequence database containing 53,216 entries. Mass tolerance on peptide precursor ions was fixed at 25 ppm and on fragment ions at 0.05 Da. The peptide charge was set to 2+ and 3+. Carbamidomethylation of cysteine was selected as a fixed modification and oxidation of methionine and de-amidation were chosen as variable modifications. False discovery rate was limited to <1% for peptide IDs after searching decoy databases.

Statistical analyses were performed using Prism 7 for windows (GraphPad Software, Inc.). Error bars represent SEM, and p-values were calculated with a two-tailed Mann–Whitney test unless stated otherwise. The key for the p-values is *P < 0.05, **P < 0.01, ***P < 0.001, ****P < 0.0001. ImageJ software was used to calculate the Spearman’s correlation (ρ) between BC6 (PrP^Sc^ aggregates) and LC3 punctae (active autophagosomes) to quantify the level of colocalisation. For immunofluorescence a minimum of 25 to maximum of 100 cells were analysed/ experiment.

### Data Availability

All data generated or analysed during this study are included in this published article (and its Supplementary Information files).

## Electronic supplementary material

Supplementary Information
