# Supplementary material for: Spermine increases acetylation of tubulins and facilitates autophagic degradation of prion aggregates
Source: Sci Rep. 2018 Jul 3;8:10004. doi: 10.1038/s41598-018-28296-y (PMC6030104; doi:10.1038/s41598-018-28296-y)

Spermine increases acetylation of tubulins and facilitates autophagic degradation of prion aggregates.

Kanchan Phadwal[1], Dominic Kurian[1], Muhammad Khalid F. Salamat[1], Vicky E. MacRae[1], Abigail B. Diack[1] and Jean C. Manson[1, 2, 3*.]

Supplementary Figure 1: PrP^{Sc} expression after 72 hrs of spermidine and spermine treatment at different dosages and MS data for acetyl-lysine pull down in SMB.s15 cells on spermine treatment (N=3).

Supplementary Figure 2: a1. Measurement of cell viability using MTT assay in SMB.s15 cells, a2. Mitochondrial membrane potential using TMRE in CAD cells. b. MS data for BC6 pull down in SMB.s15 cells with and without 10 μM CQ treatment (N=3).

Supplementary Figure 3: Full length blots shown in all the figures. Where required two different laser intensity exposure are also shown by labelling the blots as 1 and 2, 1 = intensity 5.0 - 3.0, 2 = intensity 2.0 - 1.5. Red arrows are marked to show the band used in the figures.

Supplementary Figure 1

a. Prion protein expression in SMB.s15 cells after 72hrs of spermidine and spermine treatment at dosages mentioned.

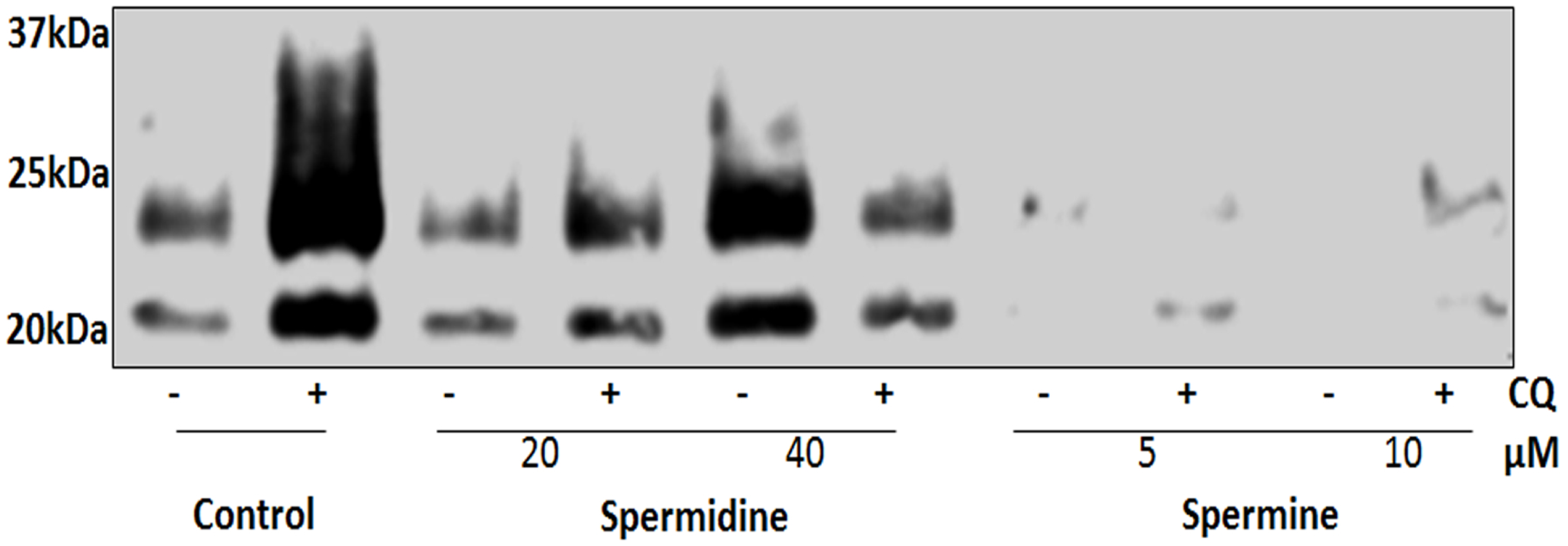

MS data for acetyl-lysine pull down from SMB.s15 cells on spermine treatment.

b. Instablue staining for total protein (1) from Acetylated-Lysine pull down products (2) from the cytoplasmic lysates of time point treated SMB cells on 5µM spermine treatment.

1. **2.**

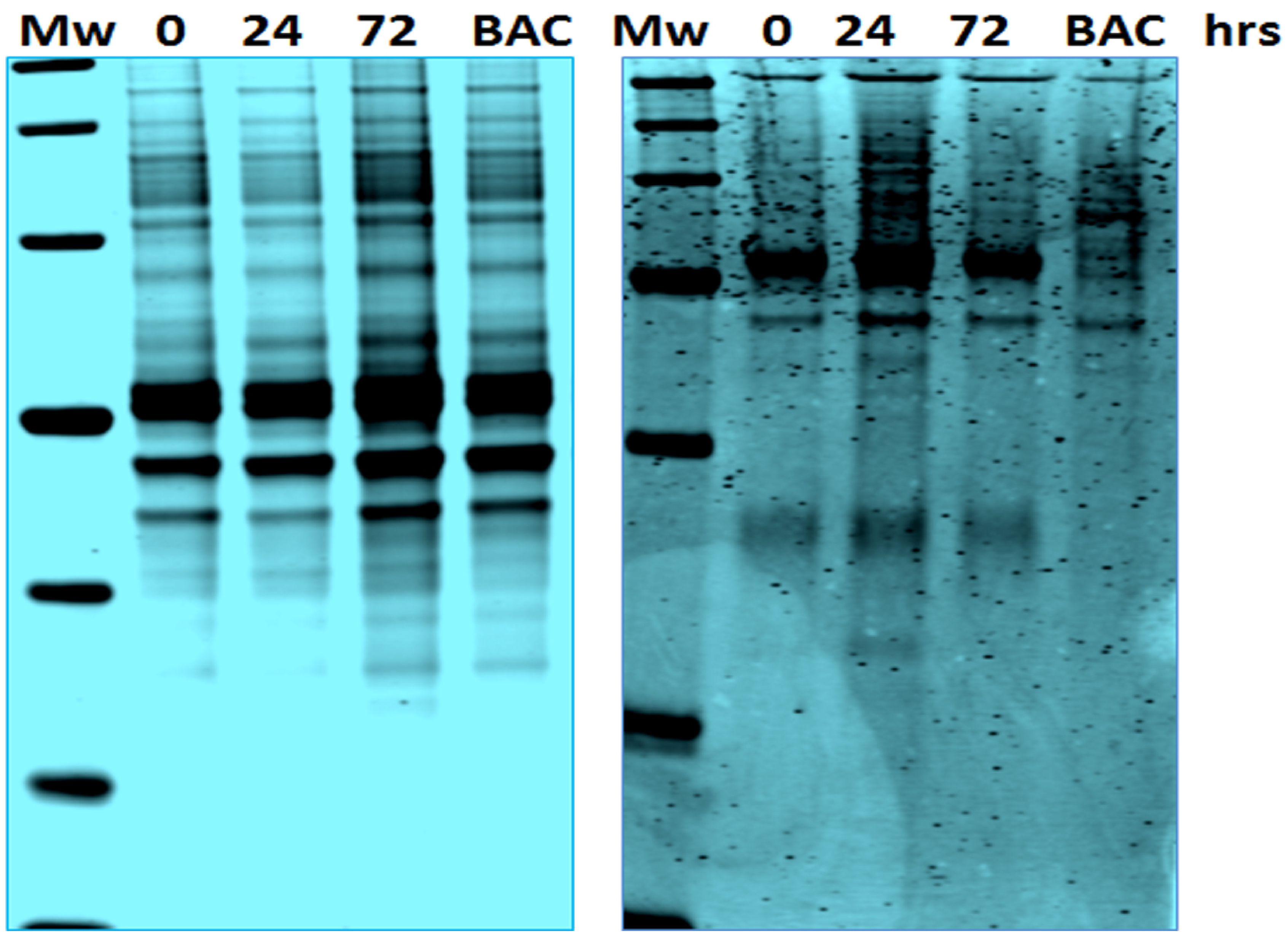

c. Mascot scores and the number of identified peptides (in the brackets) for the protein hits involved in retrograde transport obtained from the LC-MS data search after pull down with anti-Acetylated Lysine antibody.

Protein ID & UniProt Accession Number	0hr	24hr	72hr	Beads Alone Control
Tubb5, P99024	21 (1)	2993 (50)	130 (3)	Not Detected
Tubb4b, P68372	Not Detected	1919 (42)	Not Detected	Not Detected
Tuba1b, P05213	513 (9)	1808 (25)	411 (4)	Not Detected
Tuba1c, P68373	513 (9)	1698 (26)	411 (4)	Not Detected
Dync1h1, Q9JHU4	Not Detected	115 (5)	Not Detected	Not Detected

Supplementary Figure 2

a. Measurement of cell viability using MTT assay in SMB.S15 cells and mitochondrial membrane potential using TMRE in CAD cells after 72 hrs treatment with spermine.

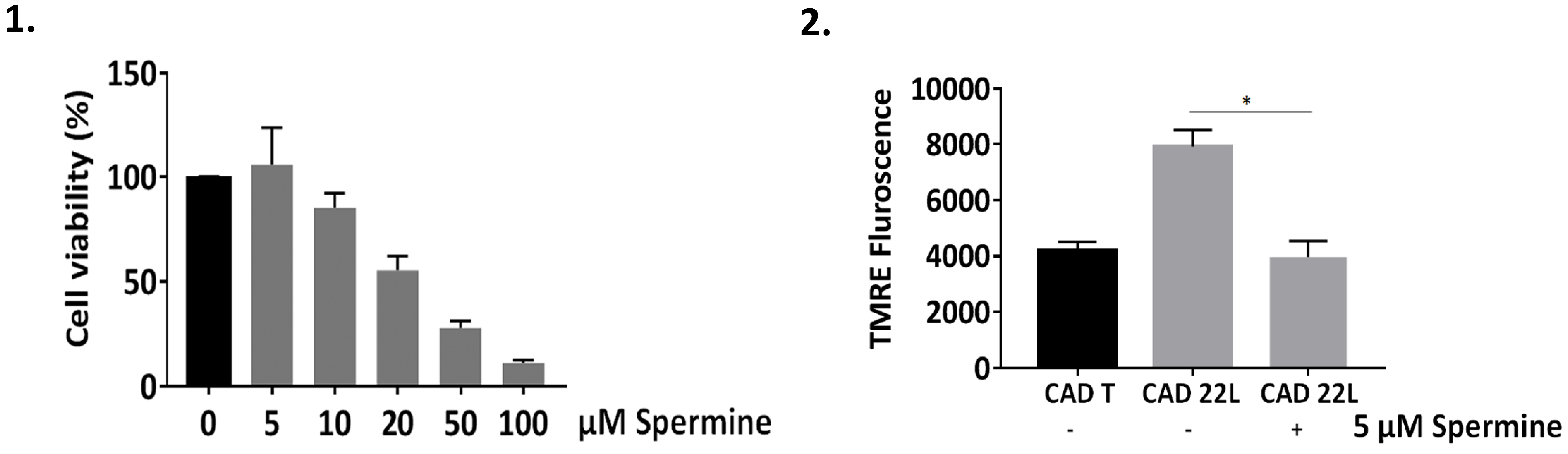

b. Mascot scores and the number of identified peptides (in the brackets), of the selected protein hits obtained from the LC-MS data after pull down with anti-prion antibody BC6.

Protein ID & UniProt Accession Number	Untreated	Chloroquine	Beads Alone Control
Tubb6, Q922F4	512 (9)	1427 (13)	Not Detected
Hsp47, P19324	Not Detected	644 (5)	Not Detected
Ubap21, Q80X50	Not Detected	243 (3)	Not Detected

Supplementary figure 3: Full length blots shown in all the figures.

Fig 1b.

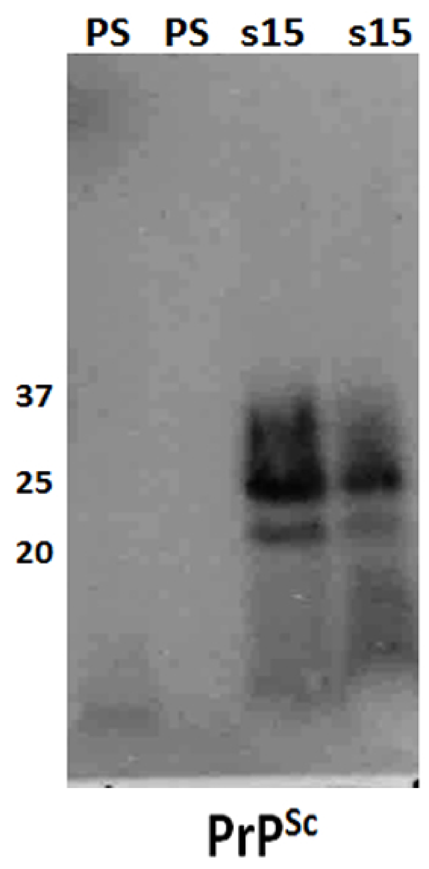

Fig 1c.

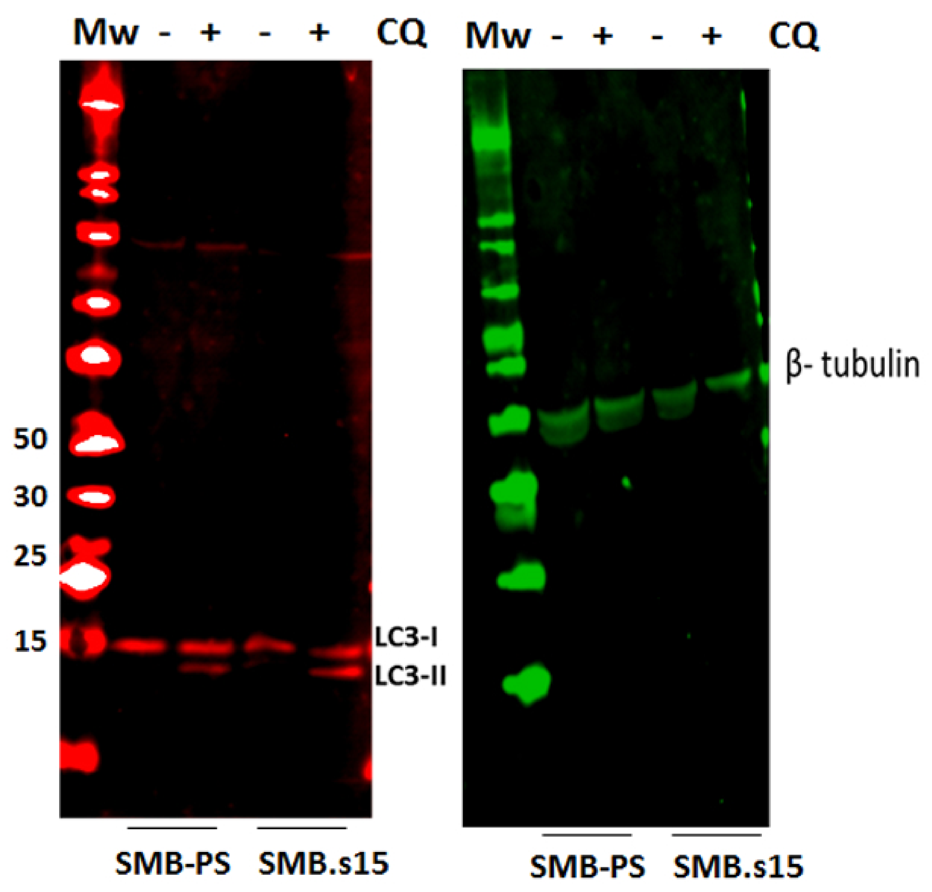

Fig 1f.

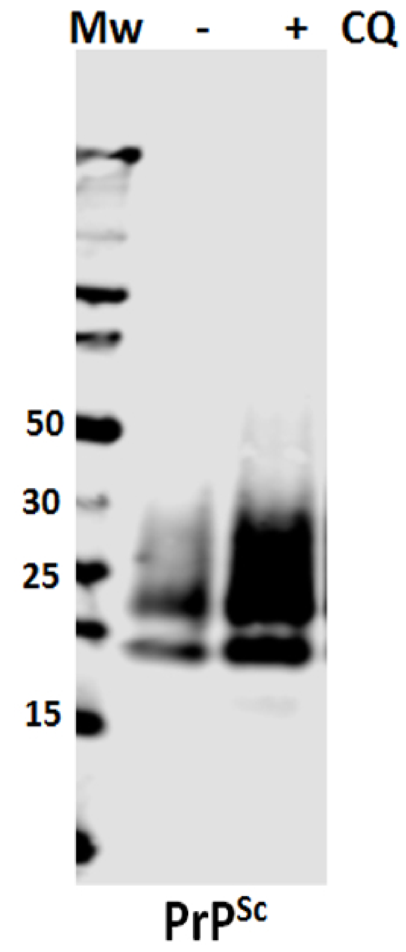

Fig 2c.

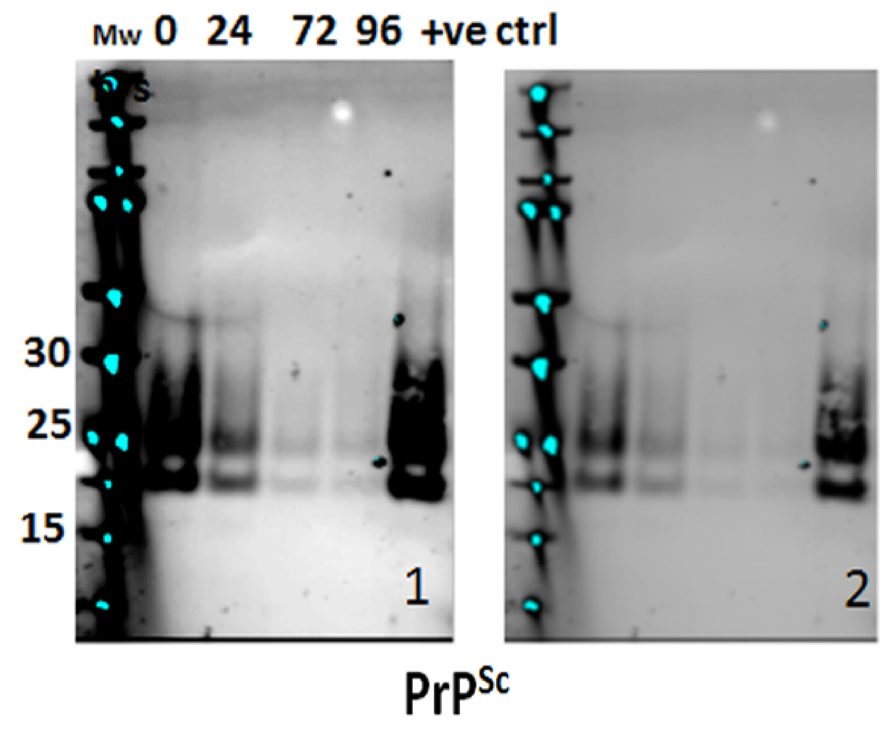

Fig 2g.

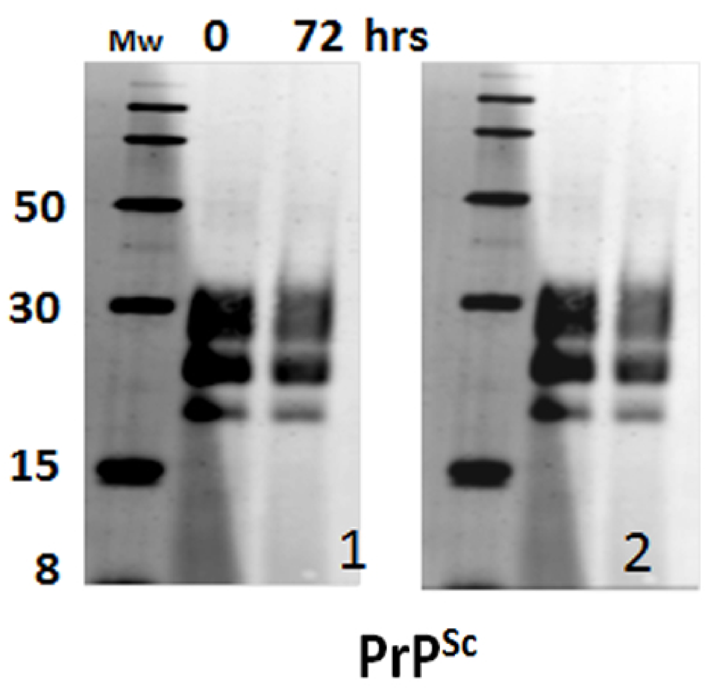

Fig 3e.

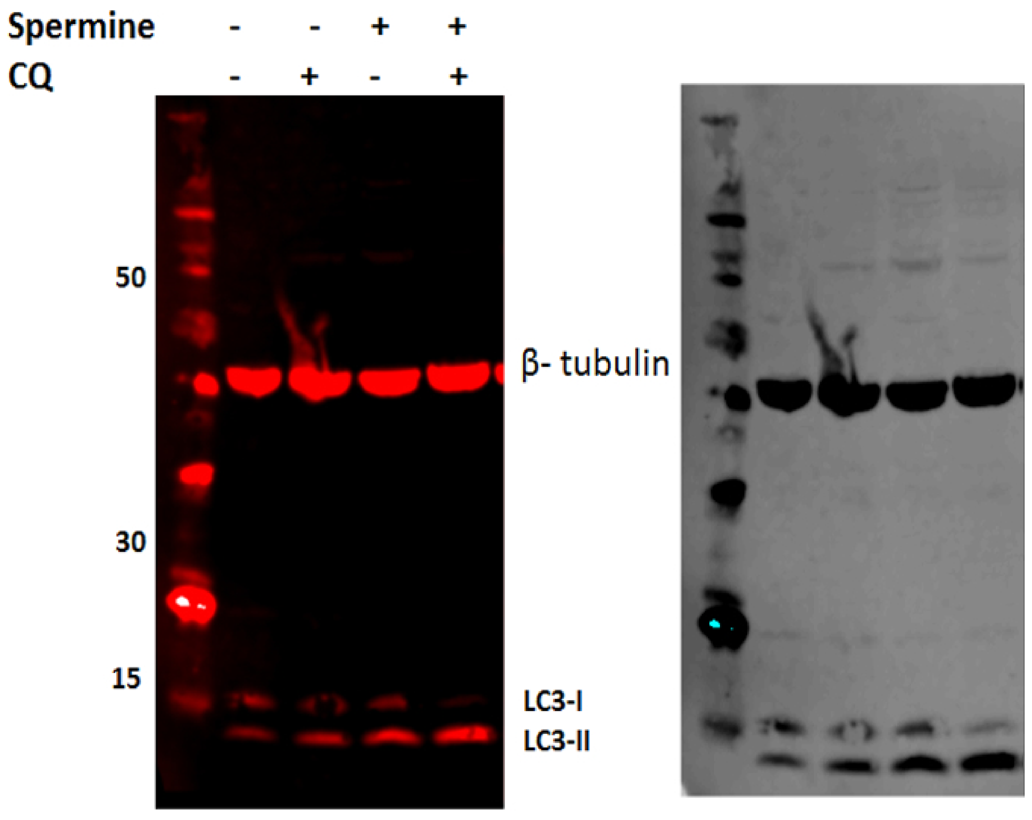

Fig 4a.

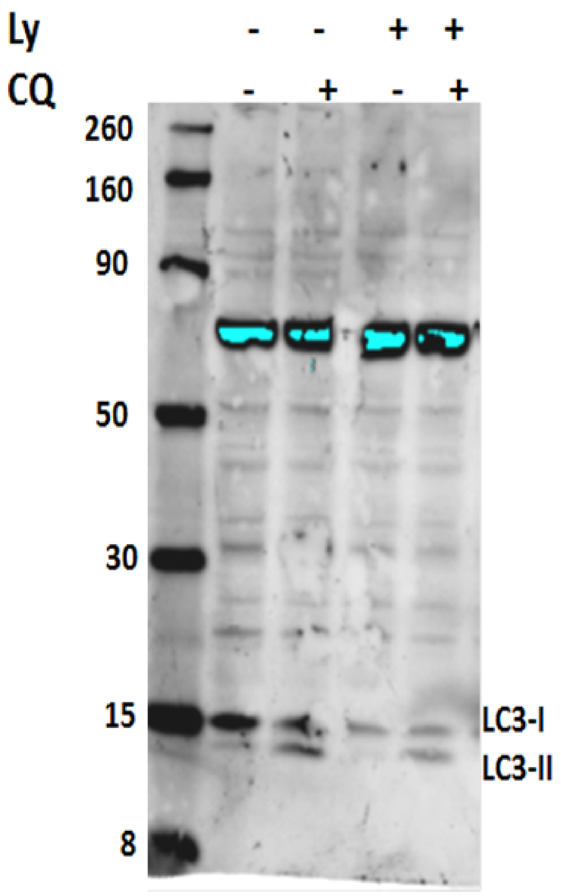

Fig 5a.

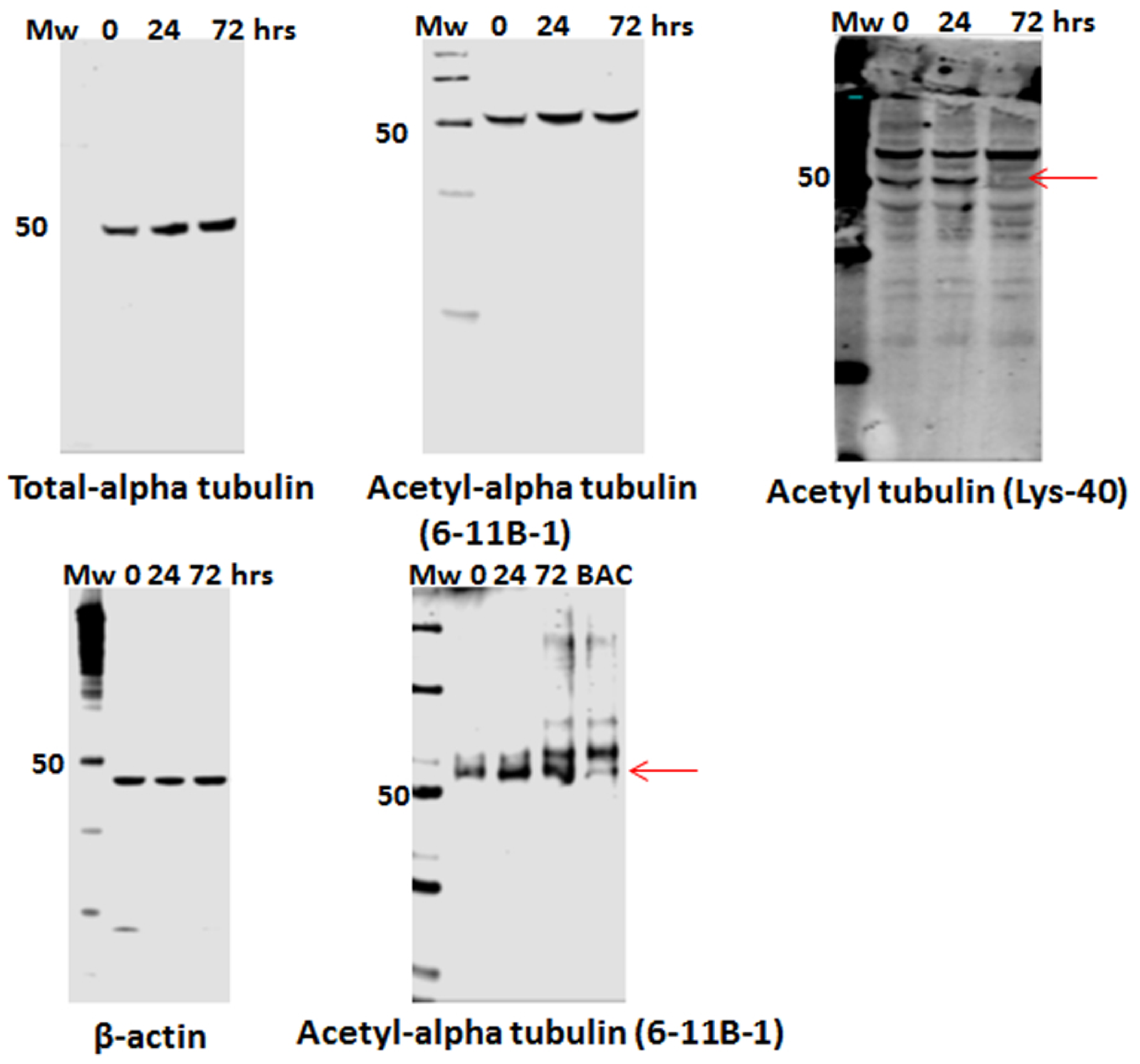

Fig 5c.

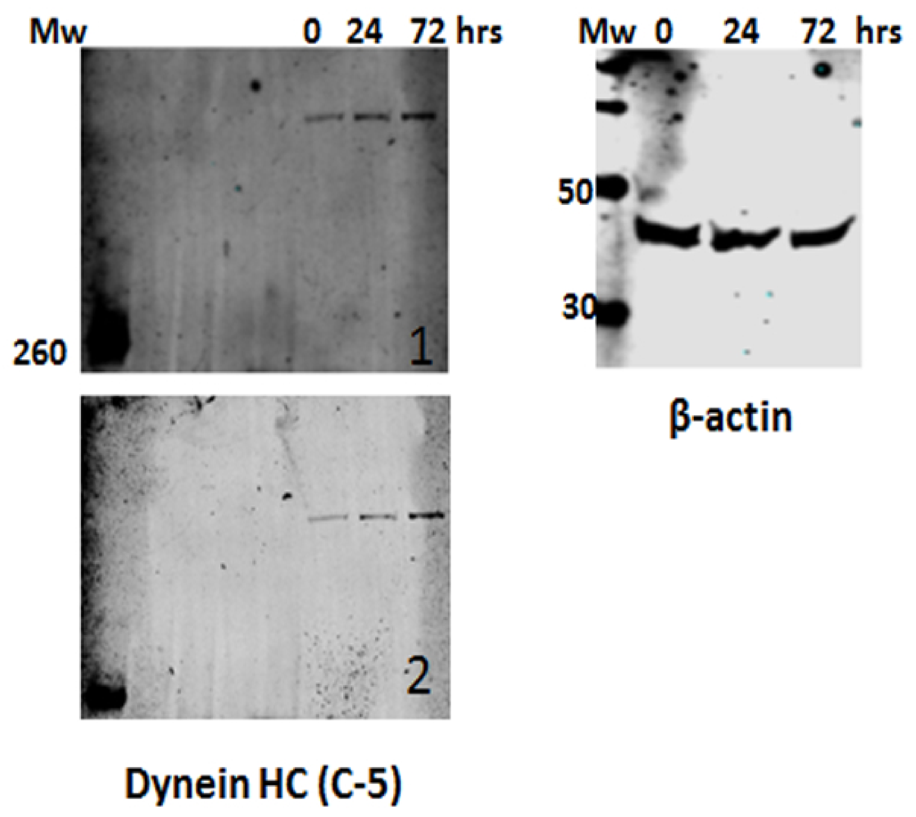

Fig 6b.

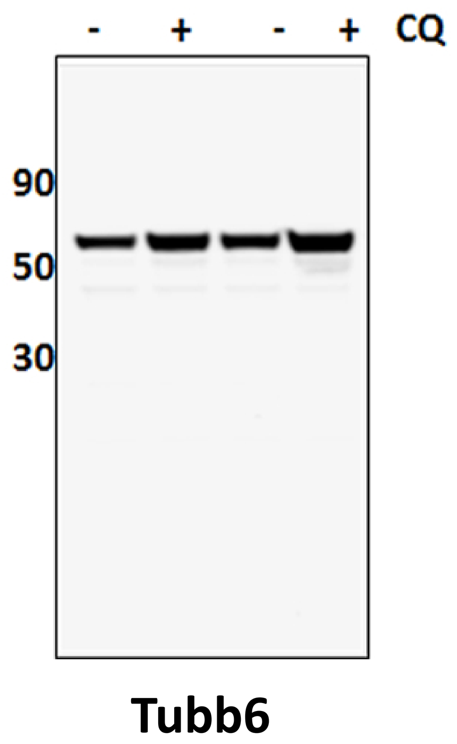

Fig 6d (3).

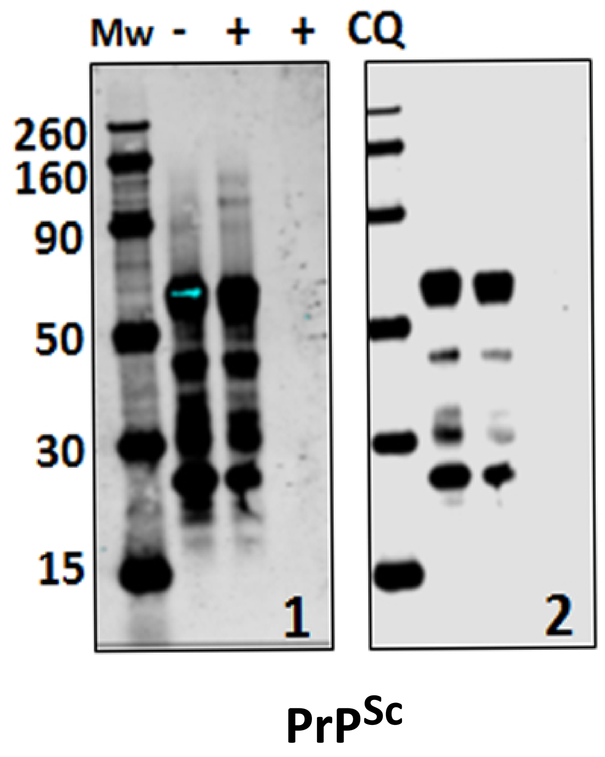

Fig 6e(2).

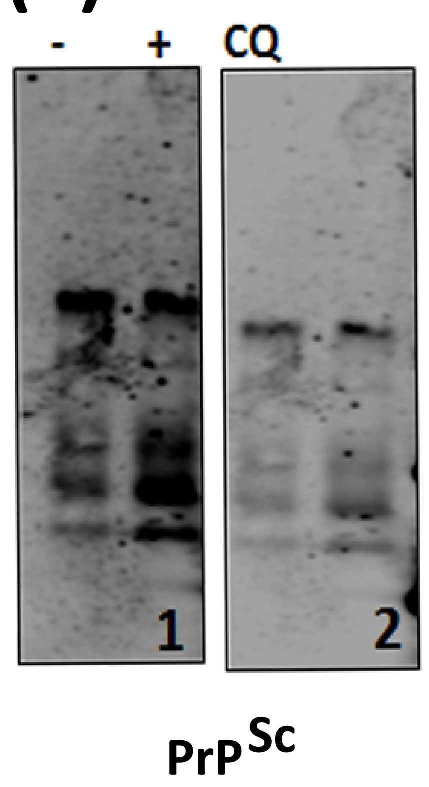

Fig 6f.

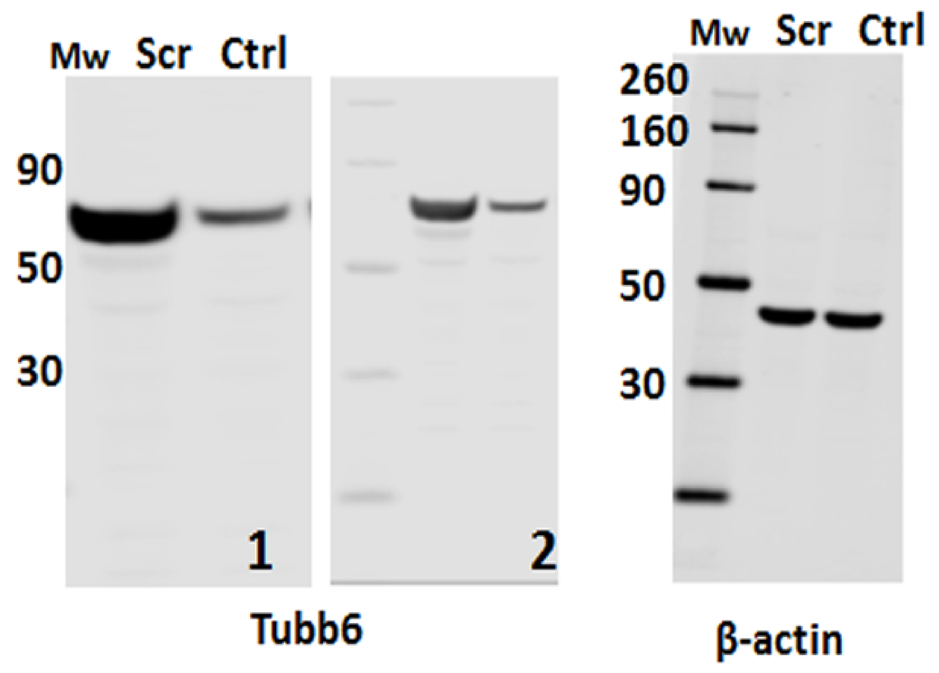

Supplement: Supplementary file 1 — Supplementary Information [file 41598_2018_28296_MOESM1_ESM.pdf]
